# Supplementary material for: Expression of a mutant CD47 protects against phagocytosis without inducing cell death or inhibiting angiogenesis
Source: Cell Rep Med. 2024 Mar 21;5(3):101450. doi: 10.1016/j.xcrm.2024.101450 (PMC10983038; doi:10.1016/j.xcrm.2024.101450)
Supplement: Document S1. Figures S1–S4 [file mmc1.pdf]

Cell Reports Medicine, Volume *5*

Supplemental information

Expression of a mutant CD47 protects against phagocytosis without inducing cell death or inhibiting angiogenesis

Lu Xu, Xiaodan Wang, Ting Zhang, Xiandi Meng, Wenjie Zhao, Chenchen Pi, and Yong-Guang Yang

SUPPLEMENTARY INFORMATION

Supplementary Figures and Legends

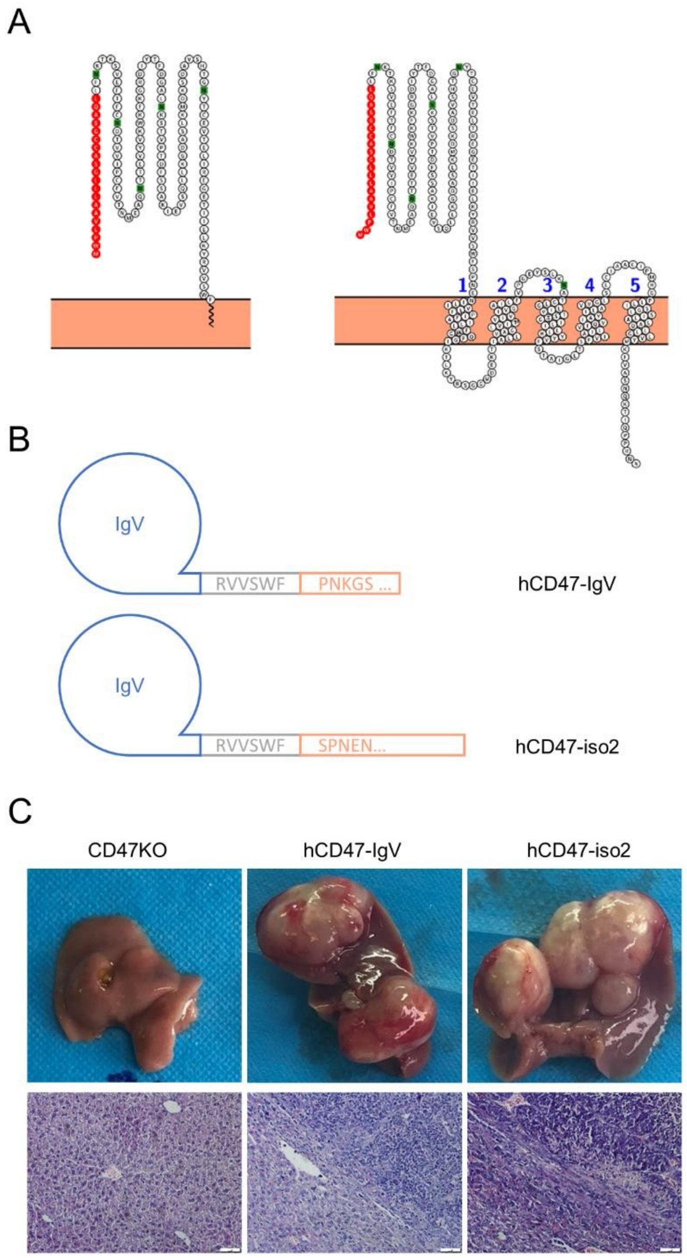

Figure S1. Schematic structure of CD47-IgV vs. CD47-iso2 and tumors developed in NCG mice receiving hCD47KO, hCD47-IgV- or hCD47-iso2-expressing Jurkat cells, related to Figure 1. (**A**). Topological structure of the CD47-IgV mutant (left) and CD47-iso2 protein (right).

CD47-IgV protein includes a signal peptide that will be cleaved off to generate the mature protein, an extracellular IgV domain and a GPI anchor; CD47- iso2 protein includes a signal peptide removed co-translationally, an extracellular IgV domain, five transmembrane regions, two extracellular loops, two intracellular loops, and an intracellular tail. (**B**). Amino acids are shown in the represent membrane-spanning segments, including the IgV domain (blue) and the rest sequences of the extracellular domain (gray), and the transmembrane domain (orange). (**C**). Autopsy and histopathologic analysis of liver samples from tumor cell-injected NCG mice**.** Livers were harvested at death or sacrifice at the end of the experiments from NCG mice injected with CD47KO (left), hCD47-IgV (middle) or hCD47-iso2 (right) Jurkat cells (as detailed in Figure 1I). Images of liver samples from representative animals are shown; scale bar, 50 μm.

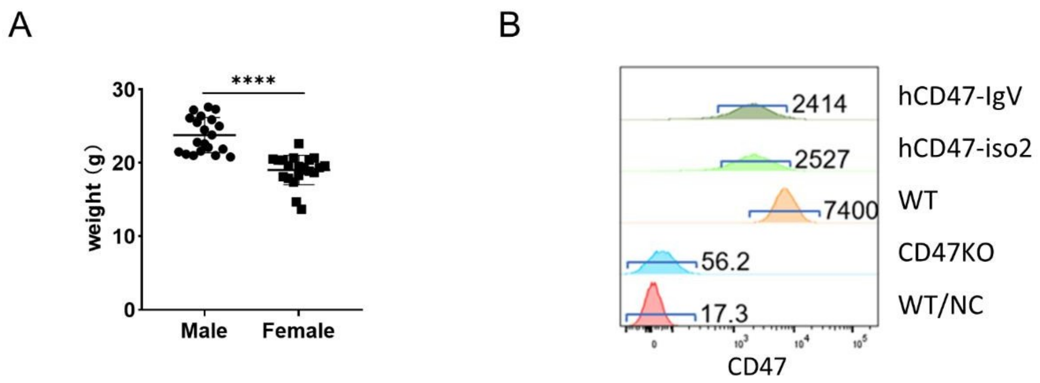

Figure S2. Bodyweights of NCG mice and CD47 expression on Jurkat cells, related to Figure 2. (**A**). Bodyweights (mean ±S.D.) of male (n=20), and female (n=21) NCG mouse recipients at the time of tumor cell injection. These mice are the same mice presented in Figure 2E. ****, $P < 0.0001$ (unpaired *t*-test). (**B**). Flow cytometry analysis of CD47 expression. Shown are CD47 staining profiles of hCD47-IgV-expressing, hCD47-iso2-expressing, WT, and CD47KO Jurkat cells. Unstained WT Jurkat cells were used as a negative control (WT/NC); numbers represent MFIs of the indicated cells.

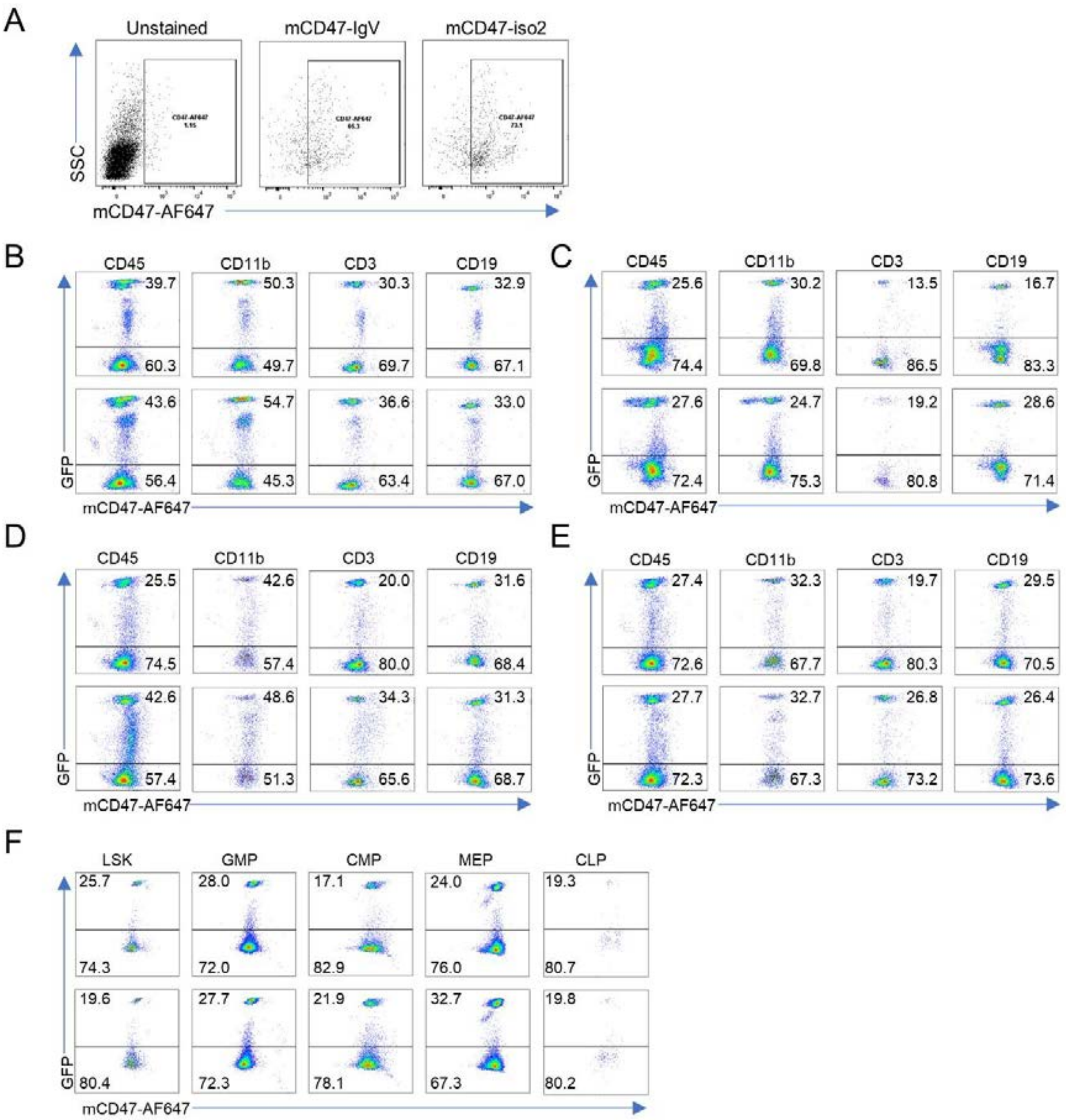

Figure S3. CD47 expression on lentivirally transduced CD47KO LSK cells and analysis of donor cell chimerism, related to Figure 3. (**A**). Flow cytometry profiles showing CD47 staining of CD47KO LSK cells transduced with mCD47-IgV or CD47-iso2 lentiviruses at 60 hours post-transduction. (**B-F**) WBCs (**B**), spleen (**C**), lymph node (**D**), and BM (**E**) cells collected from C57BL/6 recipient mice at week 14 post-transplantation were stained for CD45, CD11b, CD3 and CD19. Representative profiles showing GFP^{+} cells in total CD45^{+}, and gated CD11b^{+}, CD3^{+} and CD19^{+} cells are shown (top, mCD47-IgV group; bottom, mCD47-iso2 group). (**F**). Representative flow cytometry plots showing GFP^{+} cells in LSK, GMP, CMP, MEP and CLP in BM cells at week 14 post-transplantation (top, mCD47-IgV group; bottom, mCD47-iso2 group). Data shown were collected from the same experiments presented in Figure 3 (n=8 per group).

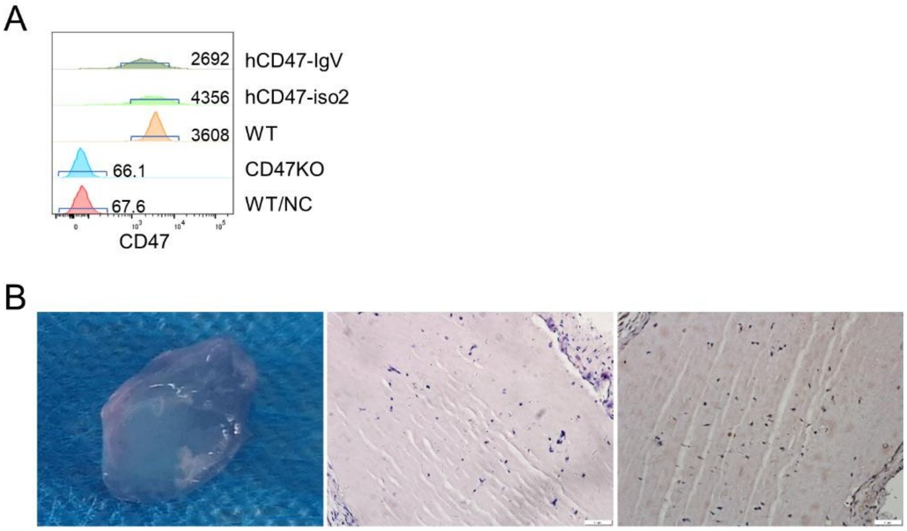

Figure S4. CD47 expression on ECs and analysis of matrigel plugs without ECs, related to Figure 4. (**A**) Shown are levels (MFIs) of CD47 expression on hCD47-IgV, hCD47-iso2 and WT ECs (EA.hy926 cells). Human CD47 antibody-stained CD47KO and unstained WT (NC) EA.hy926 cells were used as negative controls. (**B**). Matrigel plugs without ECs were injected into NCG mice and analyzed 10 days later by Autopsy (left), H&E staining (middle) and anti-hCD31 immunohistochemistry (right). Representative images are shown.
