# Supplementary material for: Expression of a mutant CD47 protects against phagocytosis without inducing cell death or inhibiting angiogenesis
Source: Cell Rep Med. 2024 Mar 21;5(3):101450. doi: 10.1016/j.xcrm.2024.101450 (PMC10983038; doi:10.1016/j.xcrm.2024.101450)
Supplement: Document S2. Article plus supplemental information [file mmc2.pdf]

Cell Reports
Medicine

Report

Expression of a mutant CD47 protects against phagocytosis without inducing cell death or inhibiting angiogenesis

Graphical abstract

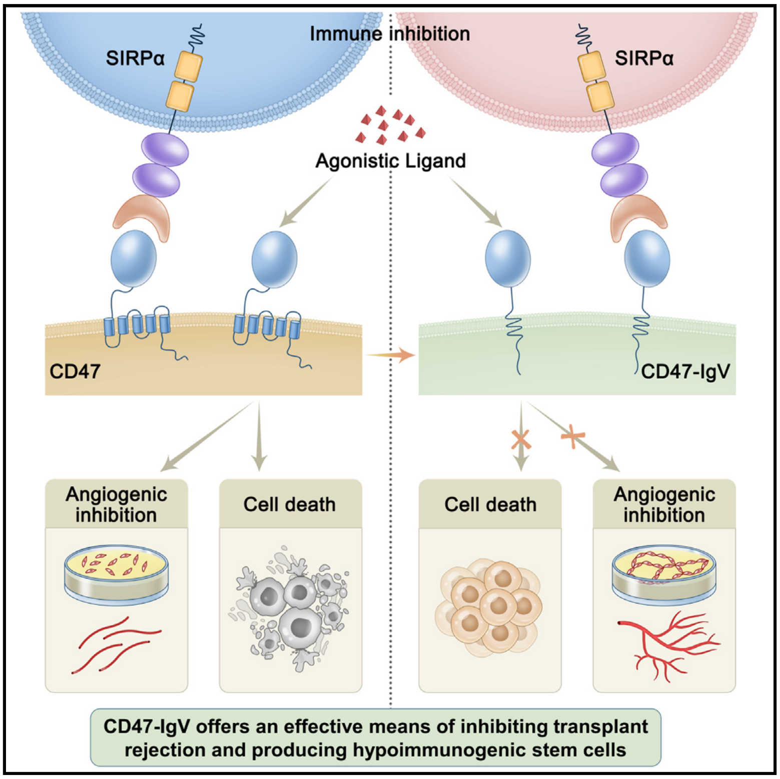

Highlights

- Transgenic expression of CD47-IgV inhibits phagocytosis as efficiently as CD47-iso2
- CD47-IgV expression fails to transmit death signals or inhibit angiogenesis
- Hematopoietic stem cells expressing CD47-IgV show no detectable functional defects

Authors

Lu Xu, Xiaodan Wang, Ting Zhang, Xiandi Meng, Wenjie Zhao, Chenchen Pi, Yong-Guang Yang

Correspondence

yongg@jlu.edu.cn

In brief

Xu and colleagues develop a mutant CD47 (CD47-IgV) by replacing the transmembrane and intracellular domains with a membrane anchor. They show that transgenic expression of CD47-IgV may inhibit phagocytosis without inducing cell death or inhibiting angiogenesis.

Xu et al., 2024, Cell Reports Medicine 5, 101450
March 19, 2024 © 2024 The Author(s).
https://doi.org/10.1016/j.xcrm.2024.101450

CellPress

Please cite this article in press as: Xu et al., Expression of a mutant CD47 protects against phagocytosis without inducing cell death or inhibiting angiogenesis, Cell Reports Medicine (2024), https://doi.org/10.1016/j.xcrm.2024.101450

Cell Reports Medicine

CellPress
OPEN ACCESS

Report

Expression of a mutant CD47 protects against phagocytosis without inducing cell death or inhibiting angiogenesis

Lu Xu,[1,2] Xiaodan Wang,[1,2] Ting Zhang,[1,2] Xiandi Meng,[1,2] Wenjie Zhao,[1,2] Chenchen Pi,[1,2] and Yong-Guang Yang[1,2,3,4,*]
[1]Key Laboratory of Organ Regeneration and Transplantation of the Ministry of Education, Institute of Immunology, First Hospital of Jilin University, Changchun, Jilin 130062, China
[2]National-Local Joint Engineering Laboratory of Animal Models for Human Disease, Jilin University, Changchun, Jilin 130062, China
[3]International Center of Future Science, Jilin University, Changchun, Jilin 130062, China
[4]Lead contact
*Correspondence: yongg@jlu.edu.cn
https://doi.org/10.1016/j.xcrm.2024.101450

SUMMARY

CD47 is a ligand of SIRPα, an inhibitory receptor expressed by macrophages, dendritic cells, and natural killer (NK) cells, and, therefore, transgenic overexpression of CD47 is considered an effective approach to inhibiting transplant rejection. However, the detrimental effect of CD47 signaling is overlooked when exploring this approach. Here, we construct a mutant CD47 by replacing the transmembrane and intracellular domains with a membrane anchor (CD47-IgV). In both human and mouse cells, CD47-IgV is efficiently expressed on the cell surface and protects against phagocytosis *in vitro* and *in vivo* but does not induce cell death or inhibit angiogenesis. Furthermore, hematopoietic stem cells expressing transgenic CD47-IgV show no detectable alterations in engraftment or differentiation. This study provides a potentially effective means of achieving transgenic CD47 expression that may help to produce gene-edited pigs for xenotransplantation and hypoimmunogenic pluripotent stem cells for regenerative medicine.

INTRODUCTION

CD47 is composed of an extracellular N-terminal immunoglobulin variable (IgV) domain, five-transmembrane helices, and an intracellular C-terminal domain.[1–3] The CD47 molecule is widely expressed on the surface of most cells and serves as a ligand of the signal regulatory protein alpha (SIRPα), an inhibitory receptor expressed on myeloid cells, including macrophages and dendritic cells (DCs), and natural killer (NK) cells.[4,5] CD47/SIRPα signaling plays an important role in controlling both innate and adaptive immune responses. By interacting with SIRPα, CD47 inhibits macrophage phagocytosis;[6] DC activation and, hence, T cell responses;[7,8] and NK cell activation.[5] Furthermore, CD47 overexpression by tumor cells serves as an important mechanism for constructing the immunosuppressive tumor microenvironment and, hence, providing an effective target for cancer immunotherapy.[9,10]

Given its strong immunoinhibitory effects, transgenic overexpression of CD47 is considered a potential strategy to prevent transplant rejection.[11,12] Previous studies have shown that macrophages mediate robust xenograft rejection predominantly due to the lack of cross-species interaction between CD47 and SIRPα, which can be effectively prevented by transgenic expression of the recipient-type CD47.[13–16] CD47 overexpression has also been reported to be effective in preventing allograft rejection,[7] offering an effective means of generating hypoimmunogenic pluripotent stem cells for regenerative medicine.[17] However, the potential adverse effect of CD47 overexpression has been largely overlooked. CD47 is not only a ligand for SIRPα, but also a receptor that mediates a variety of functions upon engagement with its ligands. It has been shown that CD47 induces cell death and inhibits angiogenesis upon ligation with its ligands, including TSP-1, soluble SIRPα, and agonistic antibodies.[18–22] CD47 signaling also inhibits insulin release from pancreatic β cells, hence affecting islet transplantation outcomes.[23] Furthermore, CD47 is an important factor mediating radiation damage[24] and ischemia-reperfusion injury.[25] Thus, there is an urgent need to develop an effective strategy that may enable CD47/SIRPα inhibitory function without the adverse effect of CD47 overexpression.

Given the critical roles of the transmembrane domains, intracellular loops, and intracytoplasmic tail in transmitting CD47 signaling,[26,27] here we sought to generate a mutant CD47 that preservers its functional interaction with SIRPα without transmitting intracellular signals by replacing the transmembrane and intracytoplasmic domains with a membrane anchor molecule, glycosylphosphatidylinositol (GPI). We found that the mutant CD47 (referred to as CD47-IgV) could be efficiently expressed on the cell surface and inhibit phagocytosis both *in vitro* and *in vivo* to a level comparable with wild-type (WT) CD47. However, unlike WT CD47, CD47-IgV does not transmit cell death signals or inhibit angiogenesis. Furthermore, hematopoietic stem cells

© 2024 The Author(s).
This is an open access article under the CC BY license (http://creativecommons.org/licenses/by/4.0/).

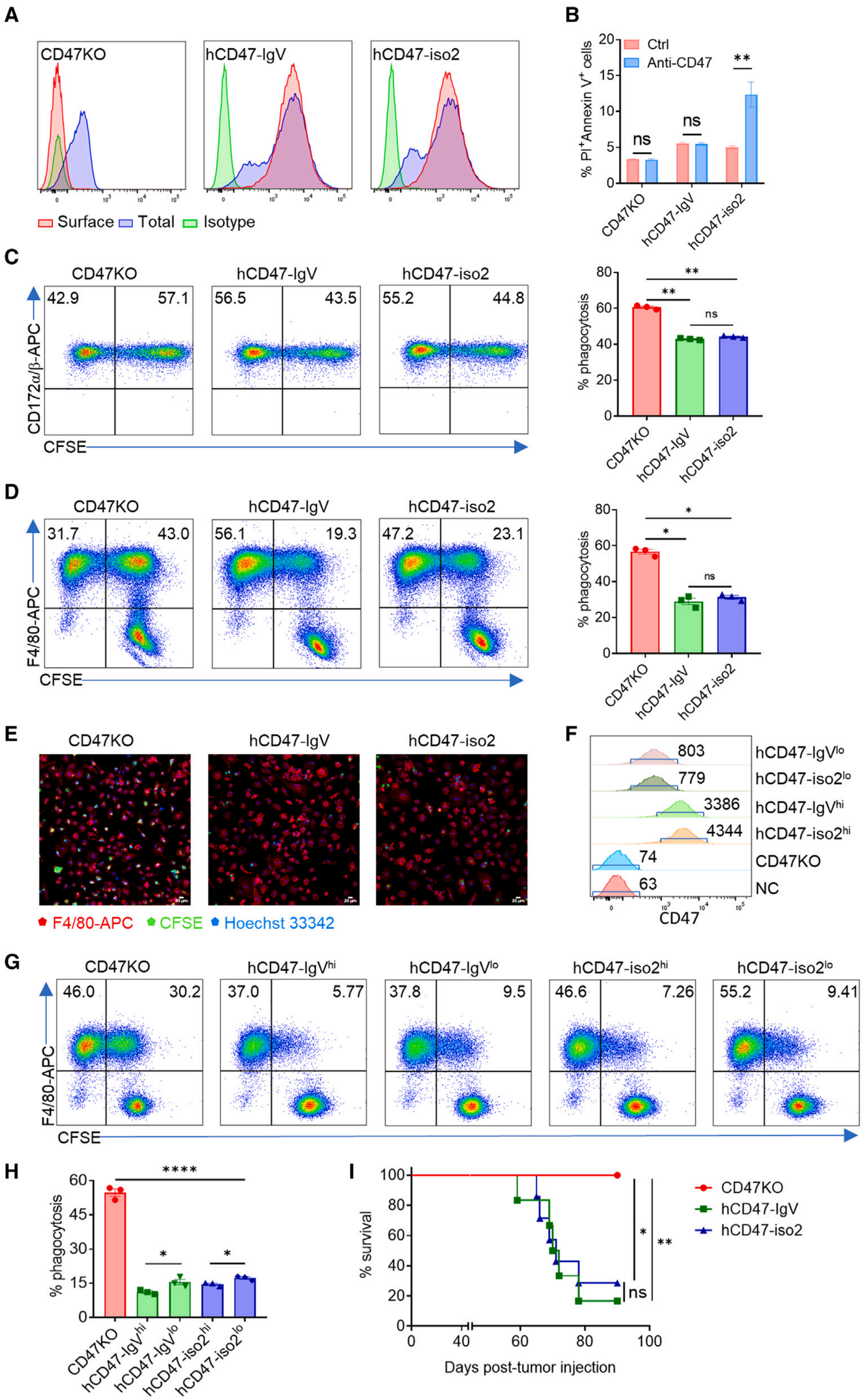

(legend on next page)

Please cite this article in press as: Xu et al., Expression of a mutant CD47 protects against phagocytosis without inducing cell death or inhibiting angiogenesis, Cell Reports Medicine (2024), https://doi.org/10.1016/j.xcrm.2024.101450

expressing CD47-IgV have no detectable defect in engraftment or blood cell differentiation. Thus, CD47-IgV may offer a strategy for transgenic CD47 overexpression to ameliorate xenograft rejection and produce hypoimmunogenic pluripotent stem cells.

RESULTS

CD47-IgV is expressed normally on the cell surface but not capable of transmitting cell death signals

A mutant human CD47 (hCD47) construct was made by replacing the hCD47 transmembrane and intracellular domains with a GPI attachment signal component as a cell membrane anchor[28] (referred to as hCD47-IgV) (Figures S1A and S1B). Because the five-transmembrane helices are involved in CD47 cell surface expression,[2,29] we first determined whether hCD47-IgV can be appropriately expressed on the cell surface. CD47 knockout (CD47KO) human Jurkat cells were transduced with lentiviral vectors encoding hCD47-IgV or hCD47 isoform 2 (hCD47-iso2; the most widely expressed CD47 isoform). Flow cytometry analysis revealed that, similar to hCD47-iso2, hCD47-IgV was efficiently expressed on the cell surface with no or minimal intracellular retention (Figure 1A). Furthermore, in agreement with the critical role of the transmembrane and intracellular domains in transmitting CD47 signaling,[26,27] incubation with an agonistic anti-CD47 antibody resulted in apoptosis in hCD47-iso2- but not hCD47-IgV-expressing cells (Figure 1B).

CD47-IgV expression inhibits phagocytosis *in vitro* and *in vivo*

We next assessed the protective effect of hCD47-IgV against phagocytosis. In the *in vitro* phagocytic assay, carboxyfluorescein succinimidyl (CFSE)-labeled CD47KO or hCD47-IgV- or hCD47-iso2-expressing Jurkat cells were incubated for 4 h with human macrophages (derived from macrophage colony stimulating factor [M-CSF]-stimulated peripheral blood mononuclear cells [PBMCs]), and phagocytosis was assessed using flow cytometry or confocal imaging by measuring the percentage of human SIRPα^+CFSE$^+$ macrophages. We found that the levels of phagocytosis of hCD47-IgV- and hCD47-iso2-expressing Jurkat cells were comparably and significantly reduced compared with CD47KO Jurkat cells (Figure 1C). Similar protective effects were also observed when these Jurkat cells were incubated with bone marrow (BM)-derived macrophages (BMDMs) of non-obese diabetic (NOD)/ShiLtJGptPrkdc^{em26Cd52}Il2rg^{em26Cd22}/Gpt (NCG) mice that express SIRPα capable of cross-reacting with hCD47.[30] Flow cytometry (Figure 1D) and confocal analysis (Figure 1E) revealed that the levels of phagocytosis of hCD47-IgV- and hCD47-iso2-expressing cells were significantly reduced compared with CD47KO Jurkat cells. In a repeated experiment, we sorted the cells with different levels of hCD47 expression and found that the magnitude of protection appeared to be correlated with the level of transgenic hCD47 expression for both hCD47-IgV- and hCD47-iso2-expressing cells (Figures 1F–1H). These results indicate that hCD47-IgV has efficiency comparable with hCD47-iso2 in inhibiting phagocytosis *in vitro*.

We then assessed potential of hCD47-IgV to inhibit rejection of Jurkat cells in immunodeficient NCG mice lacking T, B, and NK cells by comparing the tumorigenic potential of CD47KO or hCD47-IgV- or hCD47-iso2-expressing Jurkat cells. Previous studies have shown that hCD47 may functionally cross-react with NCG mouse SIRPα.[30,31] Thus, the susceptibility of these Jurkat cells to rejection by mouse macrophages is a key factor affecting tumor growth and mortality in recipient NCG mice. We observed that all NCG mice receiving CD47KO Jurkat cells intravenously (i.v.) showed long-term tumor-free survival indicative of robust rejection of CD47KO Jurkat cells (Figures 1I and S1C). However, hCD47-IgV- and hCD47-iso2-expressing Jurkat cells were comparably effective in causing tumor growth and animal death after injection into NCG mice (Figures 1I and S1C), indicating that hCD47-IgV is as effective as hCD47-iso2 in inhibiting phagocytosis *in vivo*.

To verify that the observation is not species specific, we made a mouse CD47 (mCD47) mutant by replacing the mCD47 transmembrane and intracellular domains with a GPI attachment signal anchor and assessed its expression and protection against phagocytosis. Mouse leukemic cell line A20 cells expressing mCD47-IgV or mCD47-iso2 were made by lentiviral transduction of CD47KO A20 cells that were generated by the CRISPR/Cas9 technique. Flow cytometry analysis revealed that both mCD47-IgV and mCD47-iso2 were efficiently expressed on the cell surface (Figure 2A). Furthermore, flow cytometry (Figure 2B) and confocal (Figure 2C) analysis revealed that CD47KO A20 cells were efficiently phagocytosed *in vitro* by syngeneic BALB/cAnNCrl (hereafter called BALB/c) mouse BMDMs, while both mCD47-IgV- and mCD47-iso2-expressing CD47KO A20 cells showed an equally significant decrease in phagocytosis compared with CD47KO A20 cells. mCD47-IgV- and mCD47-iso2-expressing A20 cells also showed comparably

Figure 1. Comparable protection against phagocytosis of Jurkat cells by transgenic expression of hCD47-IgV and CD47-iso2

(A) The surface and total CD47 expression of CD47KO (left), hCD47-IgV (center), and hCD47-iso2 (right).

(B) Percentages (mean ± SEM) of apoptotic (propidium iodide [PI]$^+$/Annexin V$^+$) cells of CD47KO, hCD47-IgV, and hCD47-iso2 Jurkat cells induced by an agonistic antibody (CC2C6). $^{**}p < 0.01$; NS, not significant (two-way ANOVA).

(C and D) Phagocytosis of CFSE-labeled CD47KO, hCD47-IgV, and hCD47-iso2 Jurkat cells by human macrophages (C) or NCG mouse macrophages (D). Phagocytosis was determined by flow cytometry using an anti-human CD172α/β-APC antibody (Ab) or anti-mouse F4/80-APC Ab and is presented as percentages of CFSE$^+$ human (CD172α/β^+) or mouse (F4/80$^+$) macrophages. Shown are representative flow cytometry profiles (left) and levels (right, mean ± SEM, n = 3) of phagocytosis. Representative results of three independent experiments are shown. $^*p < 0.05$, $^{**}p < 0.01$ (one-way ANOVA).

(E) Confocal images showing phagocytosis of CFSE (green)-labeled CD47KO, hCD47-IgV, or hCD47-iso2 Jurkat cells by NCG mouse macrophages (labeled with anti-F4/80-APC, red). Cell nuclei were stained with Hoechst 33342 (blue). Scale bars, 20 μm.

(F–H) Shown are levels (MFI) of CD47 expression (F) and phagocytosis by NCG mouse macrophages (G, representative flow cytometry profiles; H, percentages; mean ± SEM; n = 3) of CD47KO, hCD47-IgV^{hi}, hCD47-IgV^{lo}, hCD47-iso2^{hi}, and hCD47-iso2^{lo} Jurkat cells. $^*p < 0.05$, $^{****}p < 0.0001$ (unpaired t test).

(I) Survival curves of NCG mice that were injected (i.v.) with Jurkat cells not expressing CD47 (CD47KO; n = 6) or expressing hCD47-IgV (n = 6) or hCD47-iso2 (n = 7). $^*p < 0.05$, $^{**}p < 0.01$; NS, not significant (log rank test).

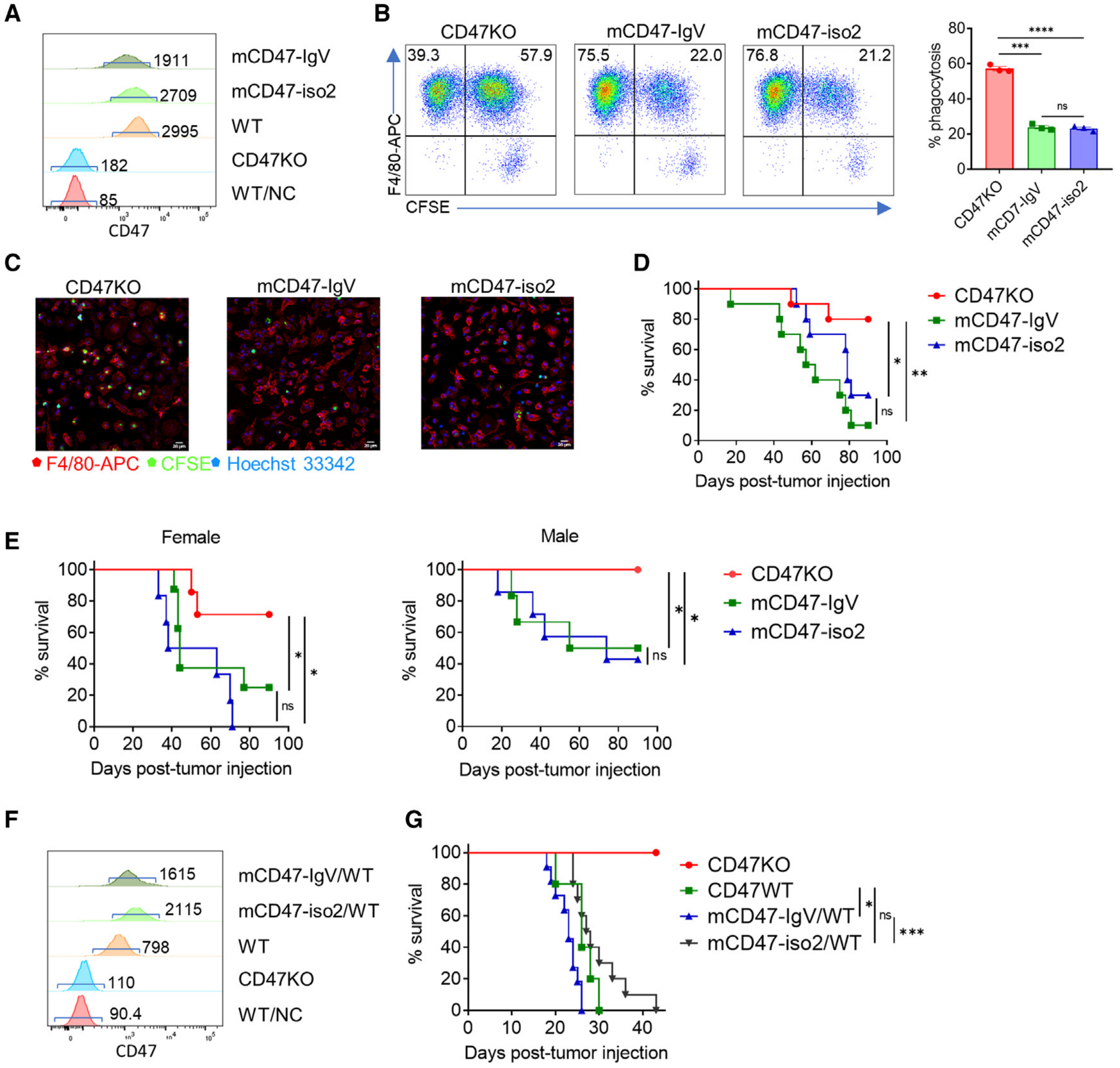

Figure 2. Mouse CD47-IgV expression efficiently inhibits phagocytosis of mouse leukemic A20 cells *in vitro* and *in vivo*

(A) CD47 expression on mCD47-IgV A20 cells, mCD47-iso2 A20 cells, WT A20 cells, and CD47KO A20 cells (the numbers indicate MFI of mouse CD47 staining). Unstained WT A20 cells (WT/NC) were used as a genative control (NC).

(B) Phagocytosis of CFSE-labeled CD47KO, mCD47-IgV, and mCD47-iso2 A20 cells by BALB/c mouse macrophages. Shown are representative flow cytometry profiles (left) and levels (right, mean ± SEMs, n = 3) of phagocytosis (i.e., percentages of macrophages that have engulfed $CFSE^+$ target cells [$CFSE^+$ cells] in mouse $F4/80^+$ macrophages). Representative results of three independent experiments are shown. ***$p < 0.001$, ****$p < 0.0001$ (one-way ANOVA).

(C) Confocal images showing phagocytosis of CFSE (green)-labeled CD47KO, mCD47-IgV, or mCD47-iso2 A20 cells by BALB/c mouse macrophages (labeled by anti-F4/80-APC, red). Cell nuclei were stained with Hoechst 33342 (blue). Scale bars, 20 μm.

(D) Survival curves of 3.5 Gy-irradiated BALB/c mice injected (i.v.) with CD47KO, mCD47-IgV, or mCD47-iso2 A20 cells (1×10^6, n = 10 per group). *$p < 0.05$, **$p < 0.01$; NS, not significant (log rank test).

(E) Survival curves of female (left) or male (right) $Rag^{-/-}$ BALB/c mice injected (i.v.) with 1×10^6 CD47KO (n = 7 for females, n = 5 for males), mCD47-IgV (n = 8 for females, n = 6 for males), or mCD47-iso2 (n = 7 for females, n = 7 for males) A20 cells. *$p < 0.05$; NS, not significant (log rank test).

(F) CD47 expression on mCD47-IgV/WT, mCD47-iso2/WT, WT, and CD47KO A20 cells (the numbers indicate MFI of mouse CD47 staining).

(G) Survival curves of 3.5-Gy-irradiated BALB/c mice injected (i.v.) with 1×10^6 CD47KO (n = 3), WT (n = 5), mCD47-IgV/WT (n = 11), or mCD47-iso2/WT (n = 10) A20 cells. *$p < 0.05$, ***$p < 0.001$; NS, not significant (log rank test).

increased leukemogenic potential compared with CD47KO A20 cells after injection into sublethally (3.5 Gy) irradiated syngeneic BALB/c mice (Figure 2D) or immunodeficient $Rag^{-/-}$ BALB/c mice (Figure 2E). Despite lacking a statistically significant difference, tumor cells expressing CD47-IgV induced higher mortality and more rapid death than those expressing CD47-iso2 in immunocompetent BALB/c mice (mortality rates and median survival times for the CD47-IgV and CD47-iso2 groups were 90% vs. 70% and 59.5 days vs. 79 days, respectively; Figure 2D), likely due to the defect of CD47-IgV in transmitting death signals (Figure 1B). Of note, there was a significant difference in body weight between female and male $Rag^{-/-}$ BALB/c mice at the time of

tumor cell injection (Figure S2A), which may explain the observed difference in mortality between female and male $Rag^{-/-}$ BALB/c mouse recipients (Figure 2E).

In these experiments, CD47-IgV- and CD47-iso2-expressing cells were generated by transgenic expression in CD47KO cells, and the levels of CD47 expression on these cells were considerably lower than the parental WT cells (Figures S2B and 2A). We next generated CD47-IgV- or CD47-iso2-expressing cells using WT A20 cells, in which WT A20 cells were transduced to express mCD47-IgV (mCD47-IgV/WT) or mCD47-iso2 (mCD47-iso2/WT). As expected, the levels of surface CD47 expression in mCD47-IgV/WT and mCD47-iso2/WT A20 cells (expressing both transgenic and endogenous CD47) were markedly higher than in WT A20 cells expressing only endogenous CD47 (Figure 2F). We then injected (i.v.) mCD47-IgV/WT- or mCD47-iso2/WT-expressing A20 cells, WT or CD47KO A20 cells (as controls) into sublethally (3.5 Gy) irradiated syngeneic BALB/c mice and followed the recipient mice for tumor formation and mortality. All mice receiving WT A20 cells, but none receiving CD47KO A20 cells, died of tumors, indicating a significant protective effect of endogenous CD47 against rejection by macrophages. Although injection of mCD47-IgV/WT or mCD47-iso2/WT A20 cells also resulted in death in all mice, significantly accelerated mortality was seen in mice receiving mCD47-IgV/WT but not mCD47-iso2/WT A20 cells compared with those receiving WT A20 cells (Figure 2G), despite the level of CD47 surface expression being higher in both CD47-IgV/WT and CD47-iso2/WT A20 cells than WT A20 cells (Figure 2F). These results confirmed that CD47-IgV expression is effective in protecting A20 cells from phagocytosis and improving survival and tumorigenesis in syngeneic mice. The more rapid death in mice receiving CD47-IgV/WT A20 cells than those injected with CD47-iso2-expressing A20 cells can presumably be attributed to the disadvantage of CD47-IgV in transmitting death signals compared with CD47-iso2 (Figure 1B).[19,20]

CD47-IgV does not affect hematopoietic stem cell function

To determine whether CD47-IgV expression may affect cell function, we determined the potential of mCD47-IgV-transduced mouse hematopoietic stem/progenitor cells (HSPCs) to engraft and differentiate in syngeneic recipients. Lineage-negative Sca-1$^+$c-kit$^+$ (LSK) HPSCs were sorted from BM cells of GFP-transgenic CD47KO C57BL/6JNifdc (C57BL/6) mice and transduced with lentiviruses encoding mCD47-IgV or mCD47-iso2. After *in vitro* culture for 60 h, the transduced cells (Figure S3A) were transplanted (i.v., 5×10^4 per mouse) into lethally irradiated syngeneic WT C57BL/6 mice (GFP$^-$) along with GFP$^-$ recipient BM cells (as carrier cells, 3×10^5 per mouse). Peripheral blood cells were collected 4, 8, and 14 weeks after transplantation and analyzed for GFP$^+$ donor cell chimerism by flow cytometry. There was no detectable difference in levels of GFP$^+$ donor total CD45$^+$, CD11b$^+$ myeloid, CD3$^+$ T, or CD19$^+$ B cells between the recipients of mCD47-IgV-transduced LSK cells and those receiving mCD47-iso2-tranduced LSK cells (Figures 3A and S3B). We also analyzed donor chimerism in the spleen (Figures 3B and S3C), lymph nodes (Figures 3C and S3D), and BM (Figures 3D and S3E) when recipient mice were sacrificed 14 weeks after transplantation and found no difference in either the levels of donor chimerism or lineage distributions between the two groups. Furthermore, BM cells from the two groups of mice had comparable levels of GFP$^+$ donor LSK, granulocyte-macrophage progenitor (GMP), common myeloid progenitor (CMP), megakaryocyte-erythrocyte progenitor (MEP), and common lymphoid progenitor (CLP) cells (Figures 3E and S3F). Together, these results indicate that CD47-IgV expression does not affect HSPC function compared with CD47-iso2 expression. Furthermore, the long-term survival of CD47KO cells with transgenic expression of mCD47-IgV provides additional evidence demonstrating the efficacy of mCD47-IgV to protect against phagocytosis.

CD47-IgV does not inhibit endothelial cell angiogenesis

To determine the ability of CD47-IgV vs. CD47-iso2 to inhibit angiogenesis, we generated CD47KO endothelial cells (ECs) using a human EC line (EA.hy926) and transduced CD47KO ECs to express hCD47-IgV or hCD47-iso2 (Figure S4A). We first compared their angiogenic potential *in vitro* using a tube formation assay, in which hCD47-IgV, hCD47-iso2, and control (WT and CD47KO) ECs were cultured with Matrigel matrix, and angiogenesis was assessed 6 h later by measuring the EC junction number (JN) and total length (TL). We found that, in agreement with the inhibitory role of CD47 in angiogenesis,[21,22] CD47KO ECs showed a significantly improved angiogenic capacity, as shown by increased JNs and TL, compared with WT ECs (Figure 4A). ECs with transgenic hCD47-IgV expression showed improved angiogenesis comparable with CD47KO ECs, while there was no detectable difference in angiogenesis between hCD47-iso2-expressing and WT ECs (Figure 4A). Furthermore, *in vitro* cell passage-induced angiogenic impairment[21] was clearly detected in WT and hCD47-iso2-expressing ECs, as shown by significantly reduced JNs and TL for passage 10 (P-10) cells compared with P-3 cells (Figure 4B). However, such an effect of cell passaging on angiogenesis was not detected in CD47KO and minimally detected in hCD47-IgV-expressing ECs (Figure 4B).

We also assessed the *in vivo* angiogenic potential of these ECs using a Matrigel plug assay. Briefly, different ECs were mixed with Matrigel and injected subcutaneously into NCG mice; Matrigel plugs were removed 10 days later and analyzed for angiogenesis by H&E and anti-hCD31 immunohistochemistry (IHC). The lack of microvessel formation in Matrigel plugs without ECs (Figure S4B) confirmed that injected ECs are responsible for neovascularization within the plugs. H&E and IHC analyses revealed that Matrigel plugs with CD47KO ECs showed a substantial increase in CD31$^+$ ECs and microvessel formation compared with Matrigel plugs with WT ECs (Figure 4C). Furthermore, plugs with hCD47-IgV ECs also showed a marked angiogenic improvement to a similar extent as those with CD47KO ECs, whereas there was no difference in EC number or microvessel formation between plugs with hCD47-iso2-expressing and WT ECs (Figure 4C). Together, both *in vitro* and *in vivo* data indicate that, different from endogenous CD47 and transgenic CD47-iso2, transgenic CD47-IgV expression on ECs has no, or at least a severely limited, inhibitory effect on angiogenesis.

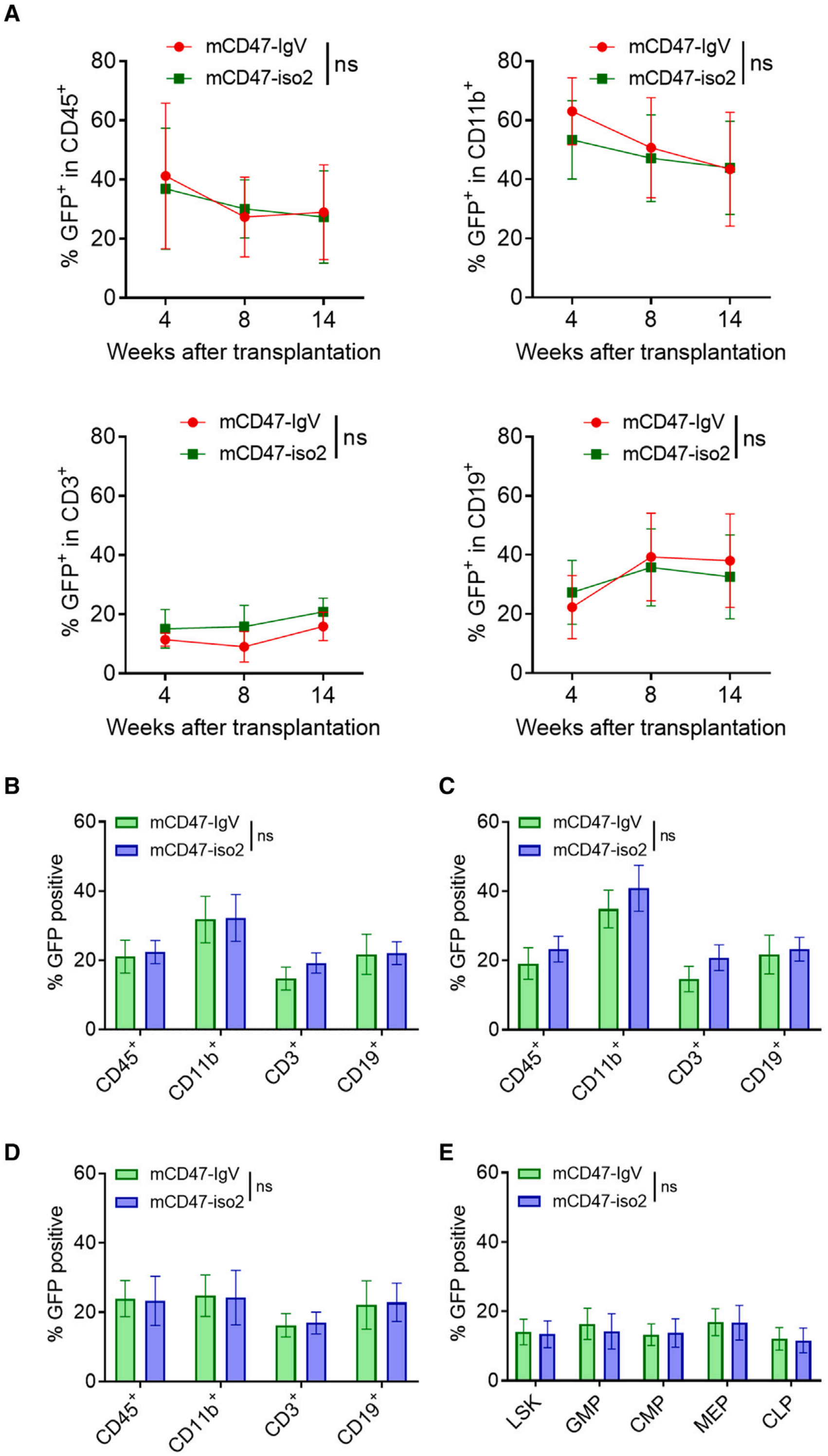

Figure 3. Comparison of engraftment and differentiation of CD47-IgV- and CD47-iso2-expressing hematopoietic stem cells

(A) WBCs were prepared at the indicated time points and analyzed for donor chimerism and lineage distribution. Shown are percentages (mean ± S.D.) of donor GFP+ cells among total CD45+ cells and gated CD11b+, CD3+, and CD19+ cells (n = 8 per group); NS, not significant (Two-way ANOVA).

(B–D) Percentages (mean ± SEM) of donor GFP+ cells among total CD45+ cells and gated CD11b+, CD3+, and CD19+ cells in the spleen (B), lymph nodes (C), and BM (D) 14 weeks after transplantation (n = 8 per group); NS, not significant (unpaired t test).

(E) Percentages (mean ± SEM, n = 8 per group) of GFP+ cells among gated LSK, GMP, CMP, MEP, and CLP cells in BM; NS, not significant (unpaired t test).

DISCUSSION

Transgenic expression of human CD47 is effective in protecting pig xenograft rejection by human macrophages.[11,12] Recently, this approach has also been applied to reduce the immunogenicity of pluripotent stem cells.[14] However, upon engagement of its agonistic ligands, CD47 also transmits apoptosis signals,[19,20] inhibits EC function and angiogenesis,[21,22] and triggers the innate alloresponse.[32] Although the IgV-like extracellular domain of CD47 is essential for its binding to SIRPα, the disulfide bond between the Ig and the multiple-membrane-spanning domains of CD47 is important in maintaining its normal topology on the cell surface and binding to SIRPα.[29,33,34] Furthermore, an engineered variant of CD47 IgV-like domain, despite its high affinity to SIRPα, was found to be incapable of triggering the inhibitory SIRPα signaling,[35] supporting a functional role of the transmembrane domains of CD47.[29] Thus, the challenge remains to generate a CD47 mutant that does not transmit intracellular signals while retaining functional interaction with SIRPα. GPI as a membrane anchor has been successfully used to achieve cell surface expression of varying fusion proteins,[36–40] including CD47.[41,42] In the present study, we constructed human and mouse CD47-IgV mutants using a method modified from previous studies[41,42] by linking the GPI attachment signal anchor to the C terminus of the CD47 IgV domain without insertion of a peptide containing charged amino acid residues and modifiable residues that may bring unpredictable structural and/or functional disturbances.[41] We show that replacement of the transmembrane and intracellular domains with GPI did not affect CD47 cell surface expression while resulting in loss of the ability to transmit CD47 signals upon engagement of agonistic anti-CD47 antibodies. Furthermore,

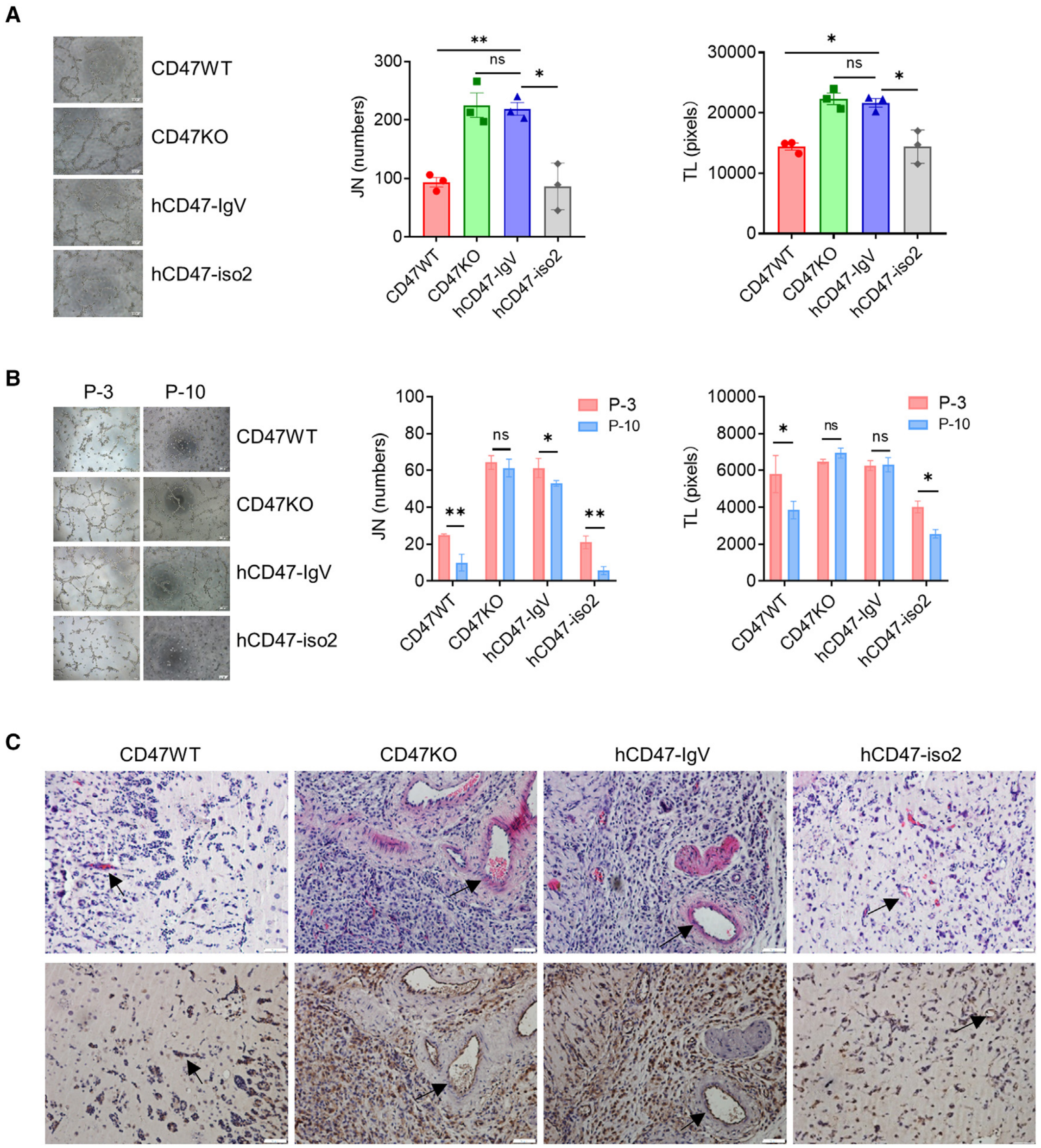

Figure 4. CD47-IgV expression does not inhibit angiogenesis *in vitro* or *in vivo*

(A) Angiogenic potential determined by tube formation assay. Shown are representative images (left; scale bars, 200 μm) and levels (mean ± SEM, n = 3) of EC junction numbers (JNs) and total length (TL) per field counted by the ImageJ plugin Angiogenesis Analyzer. Data are from one representative of two experiments. $^*p < 0.05$, $^{**}p < 0.01$; NS, not significant (one-way ANOVA).

(B) Angiogenic potential of P-3 vs. P-10 ECs determined by tube formation assay. Shown are representative images (left; scale bars, 200 μm) and levels (mean ± SEM, n = 3) of JNs and TL per field. Data are from one representative of two experiments. $^*p \leq 0.05$, $^{**}p < 0.01$; NS, not significant (unpaired t test).

(C) Analysis of neovascularization of Matrigel plugs from NCG mice by H&E (top) and anti-CD31 IHC (bottom). Arrows point to vessels; scale bars, 50 μm. The experiment (n = 3 per group) was repeated twice, and images of plugs from representative animals are shown.

CD47-IgV retains the ability to interact with SIRPα and inhibits phagocytosis to a level comparable with CD47-iso2, the most commonly expressed CD47 isoform. This study offers a CD47 mutant that lacks the multiply membrane-spanning and intracellular domains, capable of interacting with SIRPα and inhibiting phagocytosis.

The role of CD47 in promoting EC senescence and inhibiting angiogenesis[21,22] remains a major drawback to the use of organs or cells with transgenic overexpression of CD47. Here, we observed that, like CD47KO ECs, ECs expressing CD47-IgV showed significantly reduced cell senescence and improved angiogenic function compared with ECs that express

endogenous CD47 or CD47-iso2. CD47-IgV also shows defects in transmitting cell death signals compared with CD47-iso2, consistent with previous findings that the transmembrane and intracellular domains are required for transmitting CD47 signaling.[26,27] Incubation with agonistic anti-CD47 antibodies resulted in apoptosis in CD47-iso2- but not CD47-IgV-expressing cells. In agreement with the *in vitro* data, mice receiving CD47-overexpressing CD47-IgV/WT A20 cells showed significantly accelerated mortality compared with those receiving CD47-overexpressing CD47-iso2/WT A20 cells.

Previous studies have shown that the five-transmembrane helices are also involved in maintaining an appropriate CD47 topology on the cell surface, suggesting their roles in aiding CD47 cell-surface expression and/or interaction with SIRPα.[29,33,34] However, it has been reported that the IgV domain, but not the transmembrane helices, is responsible for CD47 interaction with integrins.[41] The lack of a membrane domain has been shown to decrease CD47 binding to anti-Ig domain antibodies and SIRPα-Fc, reduce CD47/SIRPα-mediated cell-cell adhesion, and affect T cell activation, Ca^{2+} response, and G_i-dependent signaling.[29,42,43] We found that mouse HSPCs with transgenic expression of CD47-IgV show no detectable defect in engraftment or differentiation after transplantation into syngeneic recipients, suggesting that transgenic overexpression of CD47-IgV in cells with expression of the endogenous CD47 is unlikely to cause significant functional defects. Thus, our results support the usefulness of the CD47-IgV mutant for transgenic overexpression, aiming to ameliorate immune responses, offering a potentially effective means of generating gene-edited pigs for xenotransplantation and hypoimmunogenic pluripotent stem cells for regenerative medicine.

Limitations of the study

The limitations of this study include the experimental models and transgene expression approach used. In this study, CD47-IgV expression was achieved by lentiviral transduction of mouse or human cells, and the effect of CD47-IgV expression was tested in cell-based *in vitro* assays and mouse models of cell transplantation. These findings, though supporting, the usefulness of the CD47-IgV mutant to improve cellular transplantation and generation of pluripotent stem cells with reduced allogenicity, provide limited information for organ xenotransplantation. Further studies involving tissue/organ transplantation from gene-edited animals (e.g., pigs) are needed to determine the advantages of CD47-IgV expression for preventing xenograft rejection.

STAR★METHODS

Detailed methods are provided in the online version of this paper and include the following:

- KEY RESOURCES TABLE
- RESOURCE AVAILABILITY
  - Lead contact
  - Materials availability
  - Data and code availability
- EXPERIMENTAL MODEL AND SUBJECT DETAILS
  - Cell culture
  - Mouse experiments
  - Mouse macrophage preparation
  - Mouse Sca-1^+c-Kit^+ $lineage^-$ (LSK) cells preparation
  - Human macrophage preparation
- METHODS DETAILS
  - Generation of cell lines
  - Flow cytometric assays
  - *In vitro* phagocytic assays
  - *In vivo* transplantation experiments
  - *In vitro* apoptosis assay
  - Hematopoietic stem cell transplantation
  - Endothelial tube formation assay
  - Matrigel plug assay
- QUANTIFICATION AND STATISTICAL ANALYSIS
  - Statistical analyses
- ADDITIONAL RESOURCES

SUPPLEMENTAL INFORMATION

Supplemental information can be found online at https://doi.org/10.1016/j.xcrm.2024.101450.

ACKNOWLEDGMENTS

This work was supported by grants from the National Key R&D Program (2021YFA1100700), NSFC (81941008 and 82171758), the Natural Science Foundation of Jilin Province, China (20200201191JC), the Department of Human Resource and Social Security of Jilin Province (2022DJ02), Science Development of Jilin Province (20230505029ZP), Natural Science Foundation of Jilin Province (20210101351JC), and the Bethune Medical Department of Jilin University (2022JBGS01).

AUTHOR CONTRIBUTIONS

Y.G.Y. and X.W. conceived and designed the research. L.X., X.W., T.Z., X.M., W.Z., and C.P. performed research and collected the data. Y.G.Y., L.X., and X.W. discussed the project, designed experiments, and/or analyzed data. Y.G.Y., L.X., and X.W. wrote the paper. All authors edited and approved the paper.

DECLARATION OF INTERESTS

The authors declare no competing interests.

Received: May 1, 2023
Revised: November 22, 2023
Accepted: February 9, 2024
Published: March 19, 2024

REFERENCES

1. Lindberg, F.P., Gresham, H.D., Schwarz, E., and Brown, E.J. (1993). Molecular cloning of integrin-associated protein: an immunoglobulin family member with multiple membrane-spanning domains implicated in alpha v beta 3-dependent ligand binding. J. Cell Biol. *123*, 485–496. https://doi.org/10.1083/jcb.123.2.485.
2. Mushegian, A. (2002). Refining structural and functional predictions for secretasome components by comparative sequence analysis. Proteins *47*, 69–74.
3. Reinhold, M.I., Lindberg, F.P., Plas, D., Reynolds, S., Peters, M.G., and Brown, E.J. (1995). In vivo expression of alternatively spliced forms of integrin-associated protein (CD47). J. Cell Sci. *108*, 3419–3425. https://doi.org/10.1242/jcs.108.11.3419.

4. Seiffert, M., Cant, C., Chen, Z., Rappold, I., Brugger, W., Kanz, L., Brown, E.J., Ullrich, A., and Bühring, H.J. (1999). Human signal-regulatory protein is expressed on normal, but not on subsets of leukemic myeloid cells and mediates cellular adhesion involving its counterreceptor CD47. Blood *94*, 3633–3643.

5. Deuse, T., Hu, X., Agbor-Enoh, S., Jang, M.K., Alawi, M., Saygi, C., Gravina, A., Tediashvili, G., Nguyen, V.Q., Liu, Y., et al. (2021). The SIRPalpha-CD47 immune checkpoint in NK cells. J. Exp. Med. *218*, e20200839. https://doi.org/10.1084/jem.20200839.

6. Oldenborg, P.A., Zheleznyak, A., Fang, Y.F., Lagenaur, C.F., Gresham, H.D., and Lindberg, F.P. (2000). Role of CD47 as a marker of self on red blood cells. Science *288*, 2051–2054. https://doi.org/10.1126/science.288.5473.2051.

7. Wang, H., Wu, X., Wang, Y., Oldenborg, P.A., and Yang, Y.G. (2010). CD47 is required for suppression of allograft rejection by donor-specific transfusion. J. Immunol. *184*, 3401–3407. https://doi.org/10.4049/jimmunol.0901550.

8. Li, Y., Zhang, M., Wang, X., Liu, W., Wang, H., and Yang, Y.G. (2020). Vaccination with CD47 deficient tumor cells elicits an antitumor immune response in mice. Nat. Commun. *11*, 581. https://doi.org/10.1038/s41467-019-14102-4.

9. Jaiswal, S., Jamieson, C.H.M., Pang, W.W., Park, C.Y., Chao, M.P., Majeti, R., Traver, D., van Rooijen, N., and Weissman, I.L. (2009). CD47 is upregulated on circulating hematopoietic stem cells and leukemia cells to avoid phagocytosis. Cell *138*, 271–285. https://doi.org/10.1016/j.cell.2009.05.046.

10. Chan, K.S., Espinosa, I., Chao, M., Wong, D., Ailles, L., Diehn, M., Gill, H., Presti, J., Jr., Chang, H.Y., van de Rijn, M., et al. (2009). Identification, molecular characterization, clinical prognosis, and therapeutic targeting of human bladder tumor-initiating cells. Proc. Natl. Acad. Sci. USA *106*, 14016–14021. https://doi.org/10.1073/pnas.0906549106.

11. Yang, Y.G., and Sykes, M. (2007). Xenotransplantation: current status and a perspective on the future. Nat. Rev. Immunol. *7*, 519–531. https://doi.org/10.1038/nri2099.

12. Perkel, J.M. (2016). Xenotransplantation makes a comeback. Nat. Biotechnol. *34*, 3–4. https://doi.org/10.1038/nbt0116-3.

13. Abe, M., Cheng, J., Qi, J., Glaser, R.M., Thall, A.D., Sykes, M., and Yang, Y.G. (2002). Elimination of porcine hemopoietic cells by macrophages in mice. J. Immunol. *168*, 621–628. https://doi.org/10.4049/jimmunol.168.2.621.

14. Wang, H., VerHalen, J., Madariaga, M.L., Xiang, S., Wang, S., Lan, P., Oldenborg, P.A., Sykes, M., and Yang, Y.G. (2007). Attenuation of phagocytosis of xenogeneic cells by manipulating CD47. Blood *109*, 836–842. https://doi.org/10.1182/blood-2006-04-019794.

15. Ide, K., Wang, H., Tahara, H., Liu, J., Wang, X., Asahara, T., Sykes, M., Yang, Y.G., and Ohdan, H. (2007). Role for CD47-SIRPalpha signaling in xenograft rejection by macrophages. Proc. Natl. Acad. Sci. USA *104*, 5062–5066. https://doi.org/10.1073/pnas.0609661104.

16. Navarro-Alvarez, N., and Yang, Y.G. (2014). Lack of CD47 on donor hepatocytes promotes innate immune cell activation and graft loss: a potential barrier to hepatocyte xenotransplantation. Cell Transplant. *23*, 345–354. https://doi.org/10.3727/096368913X663604.

17. Deuse, T., Hu, X., Gravina, A., Wang, D., Tediashvili, G., De, C., Thayer, W.O., Wahl, A., Garcia, J.V., Reichenspurner, H., et al. (2019). Hypoimmunogenic derivatives of induced pluripotent stem cells evade immune rejection in fully immunocompetent allogeneic recipients. Nat. Biotechnol. *37*, 252–258. https://doi.org/10.1038/s41587-019-0016-3.

18. Gao, A.G., and Frazier, W.A. (1994). Identification of a receptor candidate for the carboxyl-terminal cell binding domain of thrombospondins. J. Biol. Chem. *269*, 29650–29657.

19. Li, Y., Wu, Y., Federzoni, E.A., Wang, X., Dharmawan, A., Hu, X., Wang, H., Hawley, R.J., Stevens, S., Sykes, M., and Yang, Y.G. (2022). CD47 cross-dressing by extracellular vesicles expressing CD47 inhibits phagocytosis without transmitting cell death signals. Elife *11*, e73677. https://doi.org/10.7554/eLife.73677.

20. Leclair, P., Liu, C.C., Monajemi, M., Reid, G.S., Sly, L.M., and Lim, C.J. (2018). CD47-ligation induced cell death in T-acute lymphoblastic leukemia. Cell Death Dis. *9*, 544. https://doi.org/10.1038/s41419-018-0601-2.

21. Gao, Q., Chen, K., Gao, L., Zheng, Y., and Yang, Y.G. (2016). Thrombospondin-1 signaling through CD47 inhibits cell cycle progression and induces senescence in endothelial cells. Cell Death Dis. *7*, e2368. https://doi.org/10.1038/cddis.2016.155.

22. Meijles, D.N., Sahoo, S., Al Ghouleh, I., Amaral, J.H., Bienes-Martinez, R., Knupp, H.E., Attaran, S., Sembrat, J.C., Nouraie, S.M., Rojas, M.M., et al. (2017). The matricellular protein TSP1 promotes human and mouse endothelial cell senescence through CD47 and Nox1. Sci. Signal. *10*, eaaj1784. https://doi.org/10.1126/scisignal.aaj1784.

23. Ghimire, K., Kale, A., Li, J., Julovi, S.M., O'Connell, P., Grey, S.T., Hawthorne, W.J., Gunton, J.E., and Rogers, N.M. (2023). A metabolic role for CD47 in pancreatic β cell insulin secretion and islet transplant outcomes. Sci. Transl. Med. *15*, eadd2387. https://doi.org/10.1126/scitranslmed.add2387.

24. Soto-Pantoja, D.R., Miller, T.W., Pendrak, M.L., DeGraff, W.G., Sullivan, C., Ridnour, L.A., Abu-Asab, M., Wink, D.A., Tsokos, M., and Roberts, D.D. (2012). CD47 deficiency confers cell and tissue radioprotection by activation of autophagy. Autophagy *8*, 1628–1642. https://doi.org/10.4161/auto.21562.

25. Jin, G., Tsuji, K., Xing, C., Yang, Y.G., Wang, X., and Lo, E.H. (2009). CD47 gene knockout protects against transient focal cerebral ischemia in mice. Exp. Neurol. *217*, 165–170. https://doi.org/10.1016/j.expneurol.2009.02.004.

26. Lamy, L., Ticchioni, M., Rouquette-Jazdanian, A.K., Samson, M., Deckert, M., Greenberg, A.H., and Bernard, A. (2003). CD47 and the 19 kDa interacting protein-3 (BNIP3) in T cell apoptosis. J. Biol. Chem. *278*, 23915–23921. https://doi.org/10.1074/jbc.M301869200.

27. Wu, A.L., Wang, J., Zheleznyak, A., and Brown, E.J. (1999). Ubiquitin-related proteins regulate interaction of vimentin intermediate filaments with the plasma membrane. Mol. Cell *4*, 619–625. https://doi.org/10.1016/s1097-2765(00)80212-9.

28. Caras, I.W., Weddell, G.N., Davitz, M.A., Nussenzweig, V., and Martin, D.W., Jr. (1987). Signal for attachment of a phospholipid membrane anchor in decay accelerating factor. Science *238*, 1280–1283. https://doi.org/10.1126/science.2446389.

29. Rebres, R.A., Vaz, L.E., Green, J.M., and Brown, E.J. (2001). Normal ligand binding and signaling by CD47 (integrin-associated protein) requires a long range disulfide bond between the extracellular and membrane-spanning domains. J. Biol. Chem. *276*, 34607–34616. https://doi.org/10.1074/jbc.M106107200.

30. Takenaka, K., Prasolava, T.K., Wang, J.C.Y., Mortin-Toth, S.M., Khalouei, S., Gan, O.I., Dick, J.E., and Danska, J.S. (2007). Polymorphism in Sirpa modulates engraftment of human hematopoietic stem cells. Nat. Immunol. *8*, 1313–1323. https://doi.org/10.1038/ni1527.

31. Wang, C., Wang, H., Ide, K., Wang, Y., Van Rooijen, N., Ohdan, H., and Yang, Y.G. (2011). Human CD47 expression permits survival of porcine cells in immunodeficient mice that express SIRPalpha capable of binding to human CD47. Cell Transplant. *20*, 1915–1920. https://doi.org/10.3727/096368911X566253.

32. Dai, H., Friday, A.J., Abou-Daya, K.I., Williams, A.L., Mortin-Toth, S., Nicotra, M.L., Rothstein, D.M., Shlomchik, W.D., Matozaki, T., Isenberg, J.S., et al. (2017). Donor SIRPα polymorphism modulates the innate immune response to allogeneic grafts. Sci. Immunol. *2*, eaam6202. https://doi.org/10.1126/sciimmunol.aam6202.

33. Fenalti, G., Villanueva, N., Griffith, M., Pagarigan, B., Lakkaraju, S.K., Huang, R.Y.C., Ladygina, N., Sharma, A., Mikolon, D., Abbasian, M., et al. (2021). Structure of the human marker of self 5-transmembrane receptor CD47. Nat. Commun. *12*, 5218. https://doi.org/10.1038/s41467-021-25475-w.

Please cite this article in press as: Xu et al., Expression of a mutant CD47 protects against phagocytosis without inducing cell death or inhibiting angiogenesis, Cell Reports Medicine (2024), https://doi.org/10.1016/j.xcrm.2024.101450

34. Barclay, A.N., and Van den Berg, T.K. (2014). The interaction between signal regulatory protein alpha (SIRPalpha) and CD47: structure, function, and therapeutic target. Annu. Rev. Immunol. *32*, 25–50. https://doi.org/10.1146/annurev-immunol-032713-120142.

35. Ho, C.C.M., Guo, N., Sockolosky, J.T., Ring, A.M., Weiskopf, K., Özkan, E., Mori, Y., Weissman, I.L., and Garcia, K.C. (2015). Velcro" engineering of high affinity CD47 ectodomain as signal regulatory protein α (SIRPα) antagonists that enhance antibody-dependent cellular phagocytosis. J. Biol. Chem. *290*, 12650–12663. https://doi.org/10.1074/jbc.M115.648220.

36. Walter, E.I., Ratnoff, W.D., Long, K.E., Kazura, J.W., and Medof, M.E. (1992). Effect of glycoinositolphospholipid anchor lipid groups on functional properties of decay-accelerating factor protein in cells. J. Biol. Chem. *267*.

37. van den Berg, C.W., Cinek, T., Hallett, M.B., Horejsi, V., and Morgan, B.P. (1995). Exogenous glycosyl phosphatidylinositol-anchored CD59 associates with kinases in membrane clusters on U937 cells and becomes Ca(2+)-signaling competent. J. Cell Biol. *131*, 669–677. https://doi.org/10.1083/jcb.131.3.669.

38. Zhang, F., Schmidt, W.G., Hou, Y., Williams, A.F., and Jacobson, K. (1992). Spontaneous incorporation of the glycosyl-phosphatidylinositol-linked protein Thy-1 into cell membranes. Proc. Natl. Acad. Sci. USA *89*, 5231–5235. https://doi.org/10.1073/pnas.89.12.5231.

39. Huang, J.H., Getty, R.R., Chisari, F.V., Fowler, P., Greenspan, N.S., and Tykocinski, M.L. (1994). Protein transfer of preformed MHC-peptide complexes sensitizes target cells to T cell cytolysis. Immunity *1*, 607–613. https://doi.org/10.1016/1074-7613(94)90050-7.

40. Medof, M.E., Nagarajan, S., and Tykocinski, M.L. (1996). Cell-surface engineering with GPI-anchored proteins. Faseb. J. : official publication of the Federation of American Societies for Experimental Biology *10*, 574–586. https://doi.org/10.1096/fasebj.10.5.8621057.

41. Lindberg, F.P., Gresham, H.D., Reinhold, M.I., and Brown, E.J. (1996). Integrin-associated protein immunoglobulin domain is necessary for efficient vitronectin bead binding. J. Cell Biol. *134*, 1313–1322. https://doi.org/10.1083/jcb.134.5.1313.

42. McDonald, J.F., Zheleznyak, A., and Frazier, W.A. (2004). Cholesterol-independent interactions with CD47 enhance alphavbeta3 avidity. J. Biol. Chem. *279*, 17301–17311. https://doi.org/10.1074/jbc.M312782200.

43. Ticchioni, M., Raimondi, V., Lamy, L., Wijdenes, J., Lindberg, F.P., Brown, E.J., and Bernard, A. (2001). Integrin-associated protein (CD47/IAP) contributes to T cell arrest on inflammatory vascular endothelium under flow. Faseb. J. : official publication of the Federation of American Societies for Experimental Biology *15*, 341–350. https://doi.org/10.1096/fj.99-0833com.

44. Johnson, L.A., Morgan, R.A., Dudley, M.E., Cassard, L., Yang, J.C., Hughes, M.S., Kammula, U.S., Royal, R.E., Sherry, R.M., Wunderlich, J.R., et al. (2009). Gene therapy with human and mouse T-cell receptors mediates cancer regression and targets normal tissues expressing cognate antigen. Blood *114*, 535–546. https://doi.org/10.1182/blood-2009-03-211714.

45. Ran, F.A., Hsu, P.D., Wright, J., Agarwala, V., Scott, D.A., and Zhang, F. (2013). Genome engineering using the CRISPR-Cas9 system. Nat. Protoc. *8*, 2281–2308. https://doi.org/10.1038/nprot.2013.143.

46. Gao, L., Chen, K., Gao, Q., Wang, X., Sun, J., and Yang, Y.G. (2017). CD47 deficiency in tumor stroma promotes tumor progression by enhancing angiogenesis. Oncotarget *8*, 22406–22413. https://doi.org/10.18632/oncotarget.9899.

47. Schneider, C.A., Rasband, W.S., and Eliceiri, K.W. (2012). NIH Image to ImageJ: 25 years of image analysis. Nat. Methods *9*, 671–675. https://doi.org/10.1038/nmeth.2089.

48. Tan, S., Li, Y., Xia, J., Jin, C.H., Hu, Z., Duinkerken, G., Li, Y., Khosravi Maharlooei, M., Chavez, E., Nauman, G., et al. (2017). Type 1 diabetes induction in humanized mice. Proc. Natl. Acad. Sci. USA *114*, 10954–10959. https://doi.org/10.1073/pnas.1710415114.

Please cite this article in press as: Xu et al., Expression of a mutant CD47 protects against phagocytosis without inducing cell death or inhibiting angiogenesis, Cell Reports Medicine (2024), https://doi.org/10.1016/j.xcrm.2024.101450

STAR★METHODS

KEY RESOURCES TABLE

REAGENT or RESOURCE	SOURCE	IDENTIFIER
**Antibodies**		
Alexa Fluor® 647 anti-mouse CD47 antibody (clone: miap301)	Biolegend	Cat# 127510; RRID: AB_2074944
PE anti-mouse CD47 antibody (clone: miap301)	Biolegend	Cat# 127508; RRID: AB_1134117
purified anti-human CD47 antibody (clone: CC2C6)	Biolegend	Cat# 323102; RRID: AB_756132
APC Annexin V	Biolegend	Cat# 640920; RRID: AB_2561515
APC anti-human CD172α/β (SIRPα/β) antibody (clone: SE5A5)	Biolegend	Cat# 323810; RRID: AB_11219792
APC anti-mouse F4/80 antibody (clone: BM8)	Biolegend	Cat# 123116; RRID: AB_893481
Pacific Blue™ anti-mouse CD45 antibody (clone: 30-F11)	Biolegend	Cat# 103126; RRID: AB_493535
PE/Cyanine7 anti-mouse CD3ε antibody (clone: 145-2C11)	Biolegend	Cat# 100320; RRID: AB_312685
Brilliant Violet 650™ anti-mouse CD19 antibody (clone: 6D5)	Biolegend	Cat# 115541; RRID: AB_11204087
PerCP/Cyanine5.5 anti-mouse Ly-6A/E (Sca-1) antibody (clone: E13–161.7)	Biolegend	Cat# 122524; RRID: AB_893617
PE/Cyanine7 anti-mouse CD117 (c-Kit) antibody (clone:2B8)	Biolegend	Cat# 105814; RRID: AB_313223
Alexa Fluor® 700 anti-mouse lineage cocktail with isotype ctrl (clone: 17A2; RB6-8C5; RA3-6B2; Ter-119; M1/70)	Biolegend	Cat# 133313; RRID: AB_2715571
Biotin anti-mouse lineage panel (clone: 145-2C11; RB6-8C5; RA3-6B2; Ter-119; M1/70)	Biolegend	Cat#133307; RRID: AB_11124348
Alexa Fluor® 647 anti-mouse CD34 antibody (clone:SA376A4)	Biolegend	Cat# 152205; RRID: AB_2629649
Brilliant Violet 421™ anti-mouse CD135 antibody (clone:A2F10)	Biolegend	Cat# 135314; RRID: AB_2562339
PE anti-mouse CD127 (IL-7Rα) antibody (clone: SB/199)	Biolegend	Cat# 121111; RRID: AB_493510
PE anti-mouse CD11b antibody (clone: M1/70)	eBioscience	Cat# 12-0112-82; RRID: AB_2734869
Alexa Fluor™ 700 anti-mouse CD16/32 antibody (clone: 93)	eBioscience	Cat# 56-0161-82; RRID: AB_493994
Alexa Fluor® 647 anti-human CD47 (clone: B6H12)	BD Bioscience	Cat# 561249; RRID: AB_10611568
V500 Streptavidin	BD Bioscience	Cat# 561419; RRID: AB_10611863
BV711 anti-mouse CD11b (clone: M1/70)	BD Bioscience	Cat# 563168; RRID: AB_2716860
Anti-CD31 antibody	abcam	Cat# ab28364; RRID:N/A
**Bacterial and virus strains**		
TransStbl3 Chemically Competent Cell	TransGen Biotech	CD521-02
pRRLSIN.cPPT.MSCV.WPRE	Johnson et al.[44]	N/A
pSpCas9(BB)-2A-GFP (PX458)	Ran et al.[45]	Addgene PX458; Cat# 48138

(Continued on next page)

Please cite this article in press as: Xu et al., Expression of a mutant CD47 protects against phagocytosis without inducing cell death or inhibiting angiogenesis, Cell Reports Medicine (2024), https://doi.org/10.1016/j.xcrm.2024.101450

Continued

REAGENT or RESOURCE	SOURCE	IDENTIFIER
**Biological samples**		
Human peripheral blood	Healthy volunteers	N/A
**Chemicals, peptides, and recombinant proteins**		
Propidium iodide (PI)	Solarbio	P8080
Recombinant Human M-CSF Protein	R&D Systems	216-MC; GenPept: NP_757350
Recombinant Mouse M-CSF Protein	Biolegend	576408; GenPept:NM_001113530.1
Lipopolysaccharides from Escherichia coli O26:B6	Sigma	L2654
**Critical commercial assays**		
7-AAD viability staining solution	Biolegend	420404
CellTrace^{TM} CFSE Cell Proliferation Kit	ThermoFisher	C34554
Matrigel Matrix	BD Bioscience	354234
Annexin V Apoptosis Detection Kit	Biolegend	640932
**Deposited data**		
N/A	N/A	N/A
**Experimental models: Cell lines**		
Human: Jurkat line	ATCC	TIB-152
Human: 293T	ATCC	CRL-3216
Human: EA.hy926	National Collection of Authenticated Cell Cultures	GNHu39
Mouse: A20	ATCC	TIB-208
**Experimental models: Organisms/strains**		
Mouse: BALB/cAnNCrl	Beijing Vital River Laboratory Animal Technology Co., Ltd.(Beijing, China)	211
Mouse: C57BL/6Jnifdc	Beijing Vital River Laboratory Animal Technology Co., Ltd.(Beijing, China)	219
Mouse: NOD/ShiLtJGptPrkdc^{em26Cd52}Il2rge^{m26Cd22}/Gpt	Gempharmatech Co., Ltd. (Nanjing, China)	T001475
Mouse: *Rag* $^{-/-}$ BALB/c	Biogel GeneTech	N/A
Mouse: GFP-transgenic CD47 KO C57BL/6J	This paper	N/A
Oligonucleotides		
human *CD47*-specific sgRNA: CTACTGAAGTATACGTAAAG	Li et al.[19]	N/A
mouse *CD47*-specific sgRNA: TTGGCGGCGGCGCTGTTGCT	Gao et al.[46]	N/A
GPI signal sequence of decay accelerating factor	Caras IW et al.[28]	N/A
**Recombinant DNA**		
Plasmid: hCD47-IgV- pRRLSIN	This paper	N/A
Plasmid: hCD47-iso2- pRRLSIN	This paper	N/A
Plasmid: mCD47-IgV- pRRLSIN	This paper	N/A
Plasmid: mCD47-iso2- pRRLSIN	This paper	N/A
**Software and algorithms**		
ImageJ	Schneider et al.[47]	https://imagej.nih.gov/ij/
Flowjo_V10	BD Bioscience	https://www.flowjo.com/solutions/flowjo
Prism 8.0	GraphPad	https://www.graphpad.com/features

Please cite this article in press as: Xu et al., Expression of a mutant CD47 protects against phagocytosis without inducing cell death or inhibiting angiogenesis, Cell Reports Medicine (2024), https://doi.org/10.1016/j.xcrm.2024.101450

CellPress
OPEN ACCESS

RESOURCE AVAILABILITY

Lead contact

Further information and requests for resources and reagents should be directed to and will be fulfilled by the lead contact, Yong-Guang Yang (yongg@jlu.edu.cn).

Materials availability

The study did not generate new unique reagents.

Data and code availability

- All data are available in the main text.
- This paper does not report original code.
- Any additional information required to reanalyze the data reported in this work paper is available from the lead contact upon request.

EXPERIMENTAL MODEL AND SUBJECT DETAILS

Cell culture

Jurkat cells (TIB-152, ATCC) and A20 cells (TIB-208, ATCC) were cultured in RPMI 1640 medium containing 10% fetal bovine serum, 4 mM L-glutamine,100 U/ml streptomycin-penicillin. EA.hy926 (GNHu39, National Collection of Authenticated Cell Cultures) and 293T (CRL-3216, ATCC) was cultured in DMEM medium containing 10% fetal bovine serum, 4 mM L-glutamine,100 U/ml streptomycin-penicillin. The culture conditions were maintained at 37°C in a 5% CO_2 incubator of humidified.

Mouse experiments

Female or male C57BL/6Jnifdc (C57BL/6) and BALB/cAnNCrl (Balb/c) mice were purchased from Beijing Vital River Laboratory Animal Technology Co., Ltd; NOD/ShiLtJGptPrkdc^{em26Cd52}Il2rg^{em26Cd22}/Gpt (NCG) mice were purchased from Gempharmatech Co., Ltd; *Rag*$^{-/-}$ balb/c mice were purchased from Biogel GeneTech; and GFP-transgenic CD47KO C57BL/6 mice were bred in our animal facility. All animals were housed in a specific pathogen-free environment with free access to food and water and used between 8 and 10 weeks of age. Protocols involving the use of animals were reviewed and approved by the Institutional Animal Care and Use Committee of the First Hospital of Jilin University and all experiments were performed in accordance with the protocols.

Mouse macrophage preparation

Mouse macrophages were prepared from bone marrow of 8–10 weeks old female or male NCG mice or BALB/c mice by incubating with 20 ng/ml mouse M-CSF (Biolegend) for 7–9 days, followed by activation with 20 ng/ml Lipopolysaccharides (Sigma) for 8–12 h.

Mouse Sca-1$^+$c-Kit$^+$ lineage$^-$ (LSK) cells preparation

Bone marrow cells from 8 to 10 weeks old female or male CD47KO GFP-transgenic mice were depleted of lineage marker-positive (Lin$^+$) cells using a cocktail of antibodies magnetic beads. Sca-1$^+$c-Kit$^+$ lineage$^-$ (LSK) cells, which contain hematopoietic stem cells (HSCs) and multipotent progenitors (MPPs), were FACS sorted.

Human macrophage preparation

Human peripheral blood mononuclear cells (PBMCs) from healthy volunteers were isolated by Ficoll density gradient centrifugation, and the macrophages were prepared by culturing with 20 ng/ml human M-CSF (R&D Systems) for 7–9 days.

METHODS DETAILS

Generation of cell lines

CD47KO Jurkat, EA.hy926 and A20 cell lines were generated using the CRISPR/Cas9 technique as previously described.[8,46] Briefly, the three cell lines were transiently transfected with Addgene pSpCas9 (BB)-2A-GFP PX458 plasmids (#48138, a gift from Feng Zhang) containing human *CD47*-specific sgRNA (CTACTGAAGTATACGTAAAG) or mouse *CD47*-specific sgRNA (TTGGCGG CGGCGCTGTTGCT), separately,[8,19,45] and the transfected cells were sorted 48 h later by GFP expression using a BD Influx cell sorter. The cells were cultured for one week, stained with AF647-conjugated anti-human CD47 (B6H12) or anti-mouse CD47 (MIAP301) antibodies, and then sorted by three rounds to establish CD47KO cell lines. These cell lines were then used to generate human or mouse CD47-IgV- and CD47-iso2-expressing cells lines by lentiviral transduction as described.[44,48] The human and mouse CD47-IgV mutants were constructed by fusing the coding sequence of the 37 aa GPI signal sequence of decay accelerating factor (DAF)[28] with the coding sequences of human (CD47 1–137 aa) or mouse (CD47 1–161 aa) CD47-IgV, respectively, by overlap PCR,

and then sub-cloned into the pRRLSIN lentiviral vector (a gift from Steven A. Rosenberg).[44,48] The human or mouse CD47-IgV- and CD47-iso2-expressing cell lines were established by two rounds of cell sorting.

Flow cytometric assays

Single-cell suspensions were resuspended in FACS buffer, and stained with antibodies for 30 min at 4°C. The cells were then washed twice with FACS buffer and detected on a flow cytometer (BD or Cytek flow cytometer) and analyzed using FlowJo_V10 software.

In vitro phagocytic assays

To measure phagocytosis, human or mouse macrophages were cultured in 24-well plates (2×10^5/well) for 8–12 h. The culture medium was replaced with serum-free medium, and the culture was continued for 2 h, and then added with 0.5 μM carboxyfluorescein succinimidyl (CFSE)-labeled target cells (at an effect-to-target ratio of 1:4). After 4 h, the cells were harvested and stained with anti-human CD172α/β-APC or anti-mouse F4/80-APC, and observed for phagocytosis by flow cytometry or confocal macroscopy. For flow cytometric analysis, phagocytosis of target cells was defined as the percentage of CD172α/β$^+$CFSE$^+$ or F4/80$^+$CFSE$^+$ cells.

In vivo transplantation experiments

To measure *in vivo* phagocytosis of Jurkat cells, 8–10 weeks old female immunodeficient NCG mice were randomized into 3 groups, and injected with Jurkat cells expressing no CD47 (CD47KO; n = 6), hCD47-IgV (n = 6) or hCD47-iso2 (n = 7) transgenes (i.v.; 1×10^6 cells per mouse). Three sets of experiments were performed for measuring phagocytosis of A20 cells: in the first experiment, 8–10 weeks old female syngeneic BALB/c mice (preconditioned with 3.5Gy total body irradiation) were randomized into 3 groups (n = 10 for different groups), and injected with A20 cells expressing no CD47 (CD47KO), mCD47-IgV or mCD47-iso2 transgenes (i.v.; 1×10^6 cells per mouse); in the second experiment, 8–10 weeks old female BALB/c mice (preconditioned with 3.5Gy total body irradiation) were randomized into 4 groups and injected with A20 cells expressing no CD47 (CD47KO; n = 3), CD47WT (n = 5), or expressing mCD47-IgV/WT (n = 11) or mCD47-iso2/WT (n = 10) transgenes (i.v.; 1×10^6 cells per mouse); in the third experiment, *Rag*$^{-/-}$ BALB/c mice (female/male, 8–10 weeks old) were randomized into 3 groups and injected (i.v.; 1×10^6 cells per mouse) with A20 cells expressing no CD47(CD47KO, n = 7 for females; n = 5 for males), mCD47-IgV (n = 8 for females; n = 6 for males), or mCD47-iso2 (n = 7 for females; n = 7 for males). Per ethical guidelines, the transplanted mice were followed for tumor cell burden, weight loss, hindlimb paralysis, and mortality.

In vitro apoptosis assay

Jurkat cells were resuspended in complete RPMI 1640 medium without penicillin/streptomycin (P/S-free media), plated at 1×10^5 cells/well in 24-well plates, incubated at 37°C in a 5% CO_2 incubator for 2 h with or without 250 ng/mL agonist anti-CD47 antibody (CC2C6), then apoptosis was measured using an Annexin V Apoptosis Detection Kit (Biolegend) by flow cytometry according to the manufacturer's instructions (Annexin V$^+$ and PI$^+$ cells are considered dead cells).

Hematopoietic stem cell transplantation

Sca-1$^+$c-Kit$^+$ lineage$^-$ (LSK) cells were prepared from GFP-transgenic CD47KO C57BL/6 mice and transduced with mCD47-IgV- or mCD47-iso2-expressing lentiviruses (at a MOI of 10). Syngeneic recipient C57BL/6J mice (male, 8–10 weeks old) were randomized into 2 groups, conditioned with 9 Gy total body irradiation, and transplanted 6 h later with 5×10^4 transduced LSK cells and 3×10^5 carrier cells (i.e., recipient bone marrow cells) via tail vein. Levels of donor (GFP$^+$) chimerism in blood and tissues were analyzed at the indicated time points by flow cytometry.

Endothelial tube formation assay

Matrigel Matrix (BD Bioscience) was added into a 96-well plate (50 μL per well) and allowed to polymerize for 30 min at 37°C. ECs were plated at 5.5×10^4 cells/well in the 96-well plates, incubated at 37°C in a 5% CO_2 incubator for 6 h. The images were acquired after incubation. The junction number (JN) and total length (TL) in three randomly selected fields were counted by ImageJ[47] plugin Angiogenesis Analyze.

Matrigel plug assay

Matrigel plug assay was used for estimating angiogenesis *in vivo*. Briefly, 5×10^6 ECs were mixed with 400 μL Matrigel Matrix, and injected subcutaneously into 8–10 weeks old female or male NCG mice (n = 3 for every group). After 10 days, the Matrigel plugs were removed and analyzed by Hematoxylin-eosin (H&E) staining and immunohistochemistry stained with human CD31.

QUANTIFICATION AND STATISTICAL ANALYSIS

Statistical analyses

The data are presented as the mean ± SEMs from at least 3 independent experiments (Figures 1B, 1C, 1D, 1H, 2B, 3B, 3C, 3D, 3E, 4A, and 4B) or as mean ± S.D. (Figures 3A and S2). Data analysis was performed using GraphPad Prism 8.0 software. All data were

Please cite this article in press as: Xu et al., Expression of a mutant CD47 protects against phagocytosis without inducing cell death or inhibiting angiogenesis, Cell Reports Medicine (2024), https://doi.org/10.1016/j.xcrm.2024.101450

verified to follow a normal distribution and were subjected to parametric tests. Unpaired t-tests were used for comparison between two groups and one-way ANOVA or two-way ANOVA was used for comparison among more than two groups, as indicated in the figure legends. Survival data were analyzed using the log rank test. A p value of ≤ 0.05 was considered statistically significant.

ADDITIONAL RESOURCES

This study has no additional resource.

Cell Reports Medicine, Volume *5*

Supplemental information

Expression of a mutant CD47 protects against phagocytosis without inducing cell death or inhibiting angiogenesis

Lu Xu, Xiaodan Wang, Ting Zhang, Xiandi Meng, Wenjie Zhao, Chenchen Pi, and Yong-Guang Yang

SUPPLEMENTARY INFORMATION

Supplementary Figures and Legends

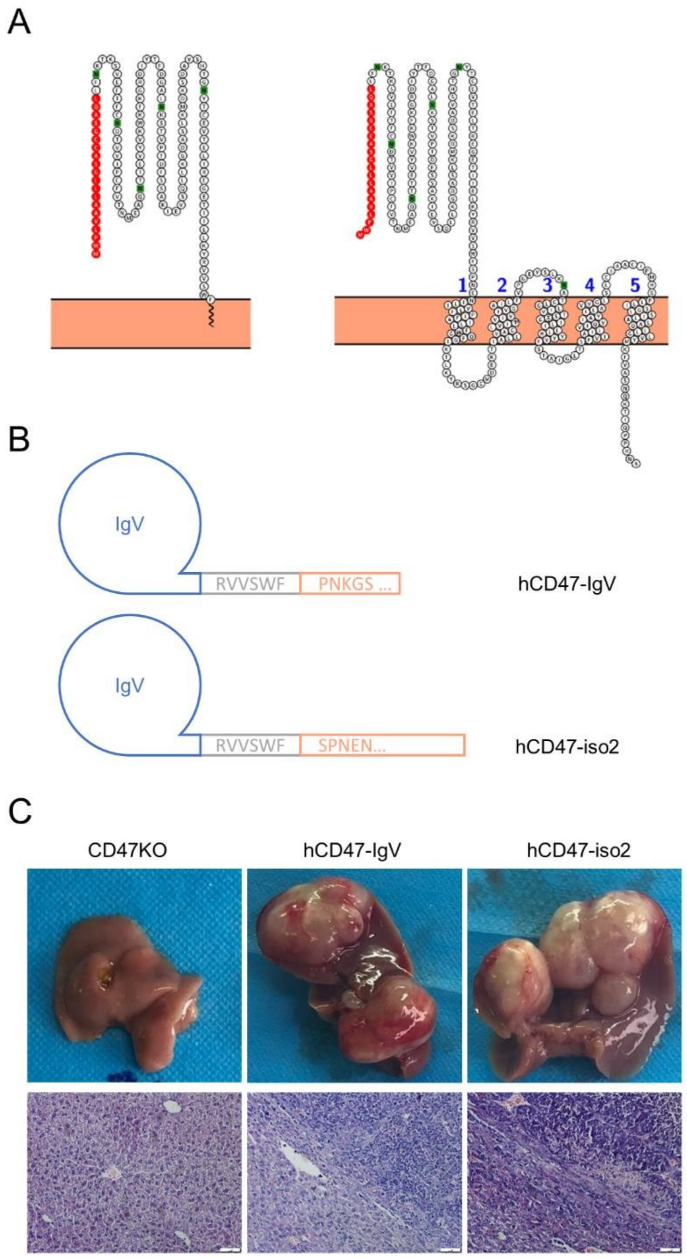

Figure S1. Schematic structure of CD47-IgV vs. CD47-iso2 and tumors developed in NCG mice receiving hCD47KO, hCD47-IgV- or hCD47-iso2-expressing Jurkat cells, related to Figure 1. (**A**). Topological structure of the CD47-IgV mutant (left) and CD47-iso2 protein (right).

CD47-IgV protein includes a signal peptide that will be cleaved off to generate the mature protein, an extracellular IgV domain and a GPI anchor; CD47- iso2 protein includes a signal peptide removed co-translationally, an extracellular IgV domain, five transmembrane regions, two extracellular loops, two intracellular loops, and an intracellular tail. (**B**). Amino acids are shown in the represent membrane-spanning segments, including the IgV domain (blue) and the rest sequences of the extracellular domain (gray), and the transmembrane domain (orange). (**C**). Autopsy and histopathologic analysis of liver samples from tumor cell-injected NCG mice**.** Livers were harvested at death or sacrifice at the end of the experiments from NCG mice injected with CD47KO (left), hCD47-IgV (middle) or hCD47-iso2 (right) Jurkat cells (as detailed in Figure 1I). Images of liver samples from representative animals are shown; scale bar, 50 µm.

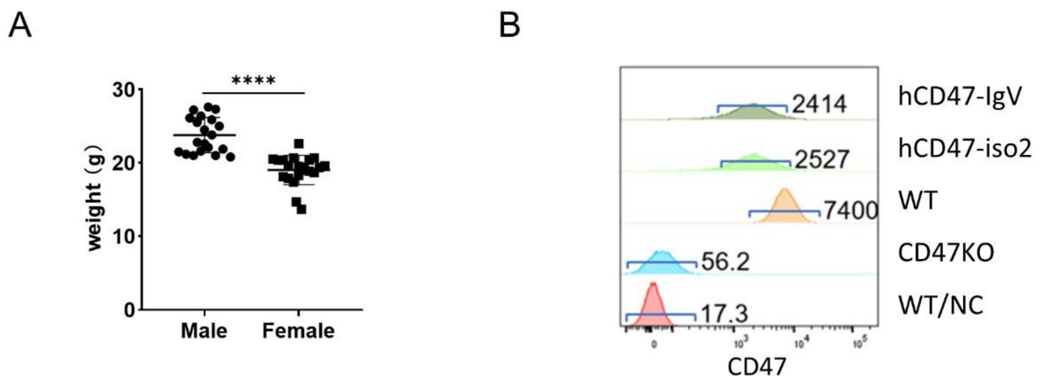

Figure S2. Bodyweights of NCG mice and CD47 expression on Jurkat cells, related to Figure 2. (**A**). Bodyweights (mean ±S.D.) of male (n=20), and female (n=21) NCG mouse recipients at the time of tumor cell injection. These mice are the same mice presented in Figure 2E. ****, $P < 0.0001$ (unpaired *t*-test). (**B**). Flow cytometry analysis of CD47 expression. Shown are CD47 staining profiles of hCD47-IgV-expressing, hCD47-iso2-expressing, WT, and CD47KO Jurkat cells. Unstained WT Jurkat cells were used as a negative control (WT/NC); numbers represent MFIs of the indicated cells.

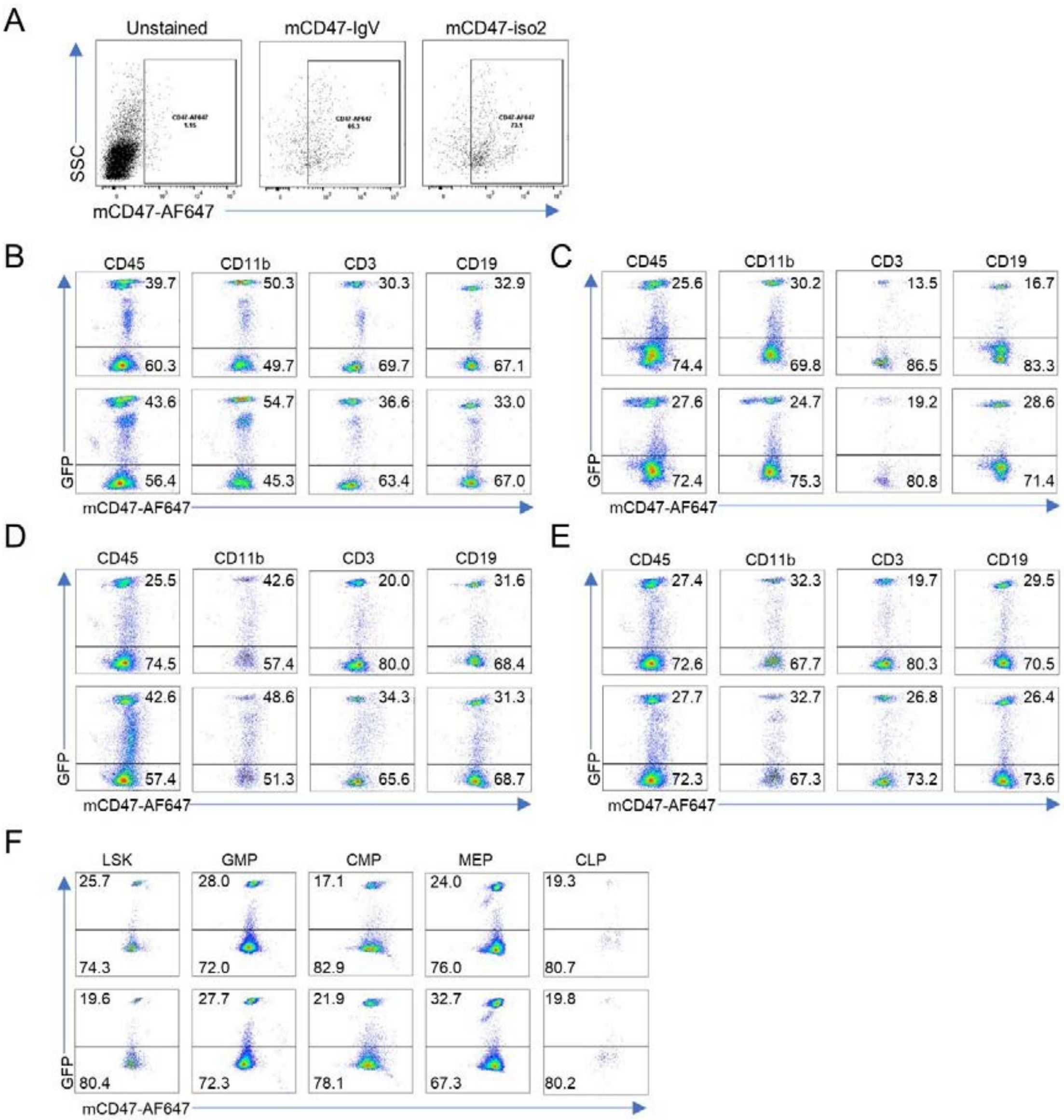

Figure S3. CD47 expression on lentivirally transduced CD47KO LSK cells and analysis of donor cell chimerism, related to Figure 3. (**A**). Flow cytometry profiles showing CD47 staining of CD47KO LSK cells transduced with mCD47-IgV or CD47-iso2 lentiviruses at 60 hours post-transduction. (**B-F**) WBCs (**B**), spleen (**C**), lymph node (**D**), and BM (**E**) cells collected from C57BL/6 recipient mice at week 14 post-transplantation were stained for CD45, CD11b, CD3 and CD19. Representative profiles showing GFP$^+$ cells in total CD45$^+$, and gated CD11b$^+$, CD3$^+$ and CD19$^+$ cells are shown (top, mCD47-IgV group; bottom, mCD47-iso2 group). (**F**). Representative flow cytometry plots showing GFP$^+$ cells in LSK, GMP, CMP, MEP and CLP in BM cells at week 14 post-transplantation (top, mCD47-IgV group; bottom, mCD47-iso2 group). Data shown were collected from the same experiments presented in Figure 3 (n=8 per group).

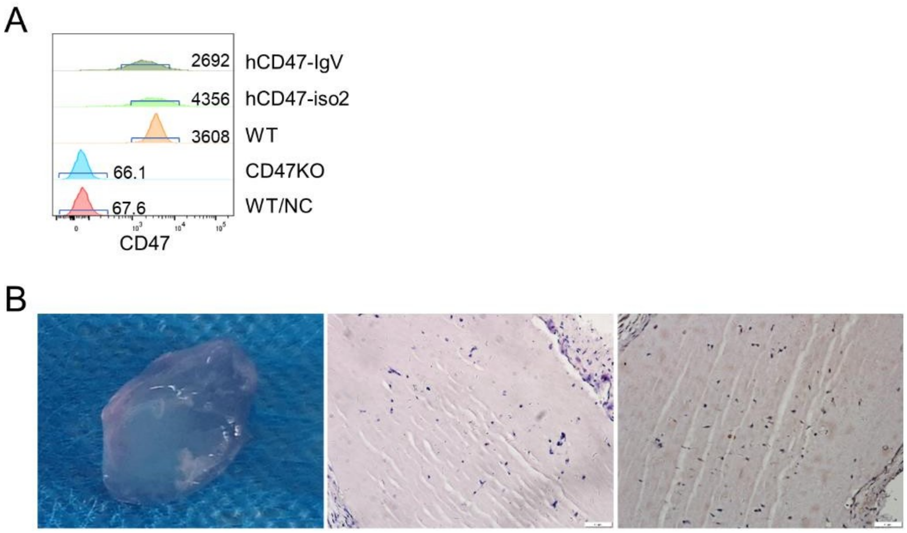

Figure S4. CD47 expression on ECs and analysis of matrigel plugs without ECs, related to Figure 4. (**A**) Shown are levels (MFIs) of CD47 expression on hCD47-IgV, hCD47-iso2 and WT ECs (EA.hy926 cells). Human CD47 antibody-stained CD47KO and unstained WT (NC) EA.hy926 cells were used as negative controls. (**B**). Matrigel plugs without ECs were injected into NCG mice and analyzed 10 days later by Autopsy (left), H&E staining (middle) and anti-hCD31 immunohistochemistry (right). Representative images are shown.
